# Trianguleniums as Optical Probes for G‐Quadruplexes: A Photophysical, Electrochemical, and Computational Study

**DOI:** 10.1002/chem.201504099

**Published:** 2016-02-16

**Authors:** Arun Shivalingam, Aurimas Vyšniauskas, Tim Albrecht, Andrew J. P. White, Marina K. Kuimova, Ramon Vilar

**Affiliations:** ^1^ Department of ChemistryImperial College London, South KensingtonLondonSW7 2AZUK

**Keywords:** DNA, optical probes, nucleic acids, quadruplexes, triangulenium

## Abstract

Nucleic acids can adopt non‐duplex topologies, such as G‐quadruplexes in vitro. Yet it has been challenging to establish their existence and function in vivo due to a lack of suitable tools. Recently, we identified the triangulenium compound **DAOTA‐M2** as a unique fluorescence probe for such studies. This probe's emission lifetime is highly dependent on the topology of the DNA it interacts with opening up the possibility of carrying out live‐cell imaging studies. Herein, we describe the origin of its fluorescence selectivity for G‐quadruplexes. Cyclic voltammetry predicts that the appended morpholino groups can act as intra‐ molecular photo‐induced electron transfer (PET) quenchers. Photophysical studies show that a delicate balance between this effect and inter‐molecular PET with nucleobases is key to the overall fluorescence enhancement observed upon nucleic acid binding. We utilised computational modelling to demonstrate a conformational dependence of intra‐molecular PET. Finally, we performed orthogonal studies with a triangulenium compound, in which the morpholino groups were removed, and demonstrated that this change inverts triangulenium fluorescence selectivity from G‐quadruplex to duplex DNA, thus highlighting the importance of fine tuning the molecular structure not only for target affinity, but also for fluorescence response.

## Introduction

Guanine‐rich nucleic acids can fold into non‐canonical structures termed G‐quadruplexes. Composed of helically stacked G‐tetrads, these quadruple stranded structures have attracted a great deal of attention. Mounting evidence suggests they play key roles in biologically important processes ranging from telomerase dysfunction to regulation of gene expression.^1^, ^2^ Consequently, selective disruption of their normal function could elicit therapeutic effects. To this end, numerous small molecules have been developed to target G‐quadruplexes.^3^, ^4^, ^5^ However, given that the majority of small molecules bind non‐covalently and that G‐quadruplexes arise from reversible inter‐/intra‐molecular forces, it has been challenging to determine the extent to which these compounds interact with G‐quadruplexes in live cells.^6^, ^7^, ^8^

**Scheme 1:**
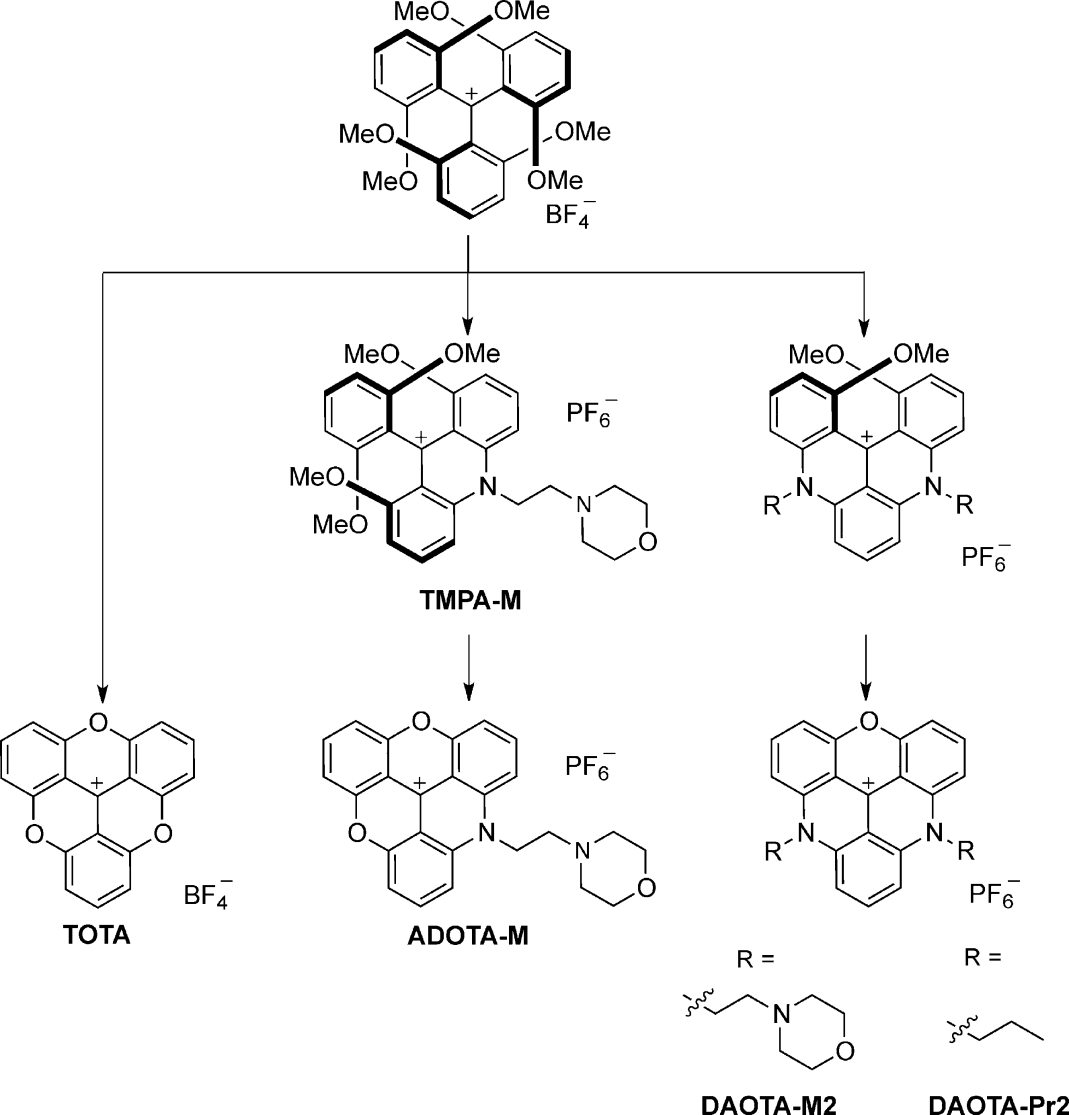
Synthetic route and chemical structure of trianguleniums studied in this work. (i) Py**⋅**HCl, 200 °C, 1 h; (ii) 4‐(2‐aminoethyl)morpholine, *N*‐methyl‐2‐pyrrolidone (NMP), room temperature, 2 h then NH_4_PF_6_ (aq.); (iii) RNH_2_, NMP, 110 °C, 2 h then NH_4_PF_6_ (aq.).

One method that allows direct yet non‐invasive detection of interactions with G‐quadruplexes is fluorescence spectroscopy and microscopy. Small molecules that have emissive properties that change upon binding to G‐quadruplexes offer an ideal means of probing the relationship between in vitro selectivity and cellular engagement.^9^, ^10^ In this regard, we recently reported a small fluorescent molecule (**DAOTA‐M2**, Scheme 1), the fluorescence lifetime of which is significantly altered upon binding to different nucleic acid topologies.^11^ This new optical probe possesses excellent cellular uptake properties (nuclear localisation, DNA binding, as well as low photo‐/cyto‐toxicity) and, promisingly, could differentiate between G‐quadruplexes and other nucleic acid topologies through fluorescence lifetime measurements in vitro. Furthermore, imaging studies by using fluorescence lifetime imaging microscopy (FLIM) suggested that the probe also targets G‐quadruplexes in live cells. The advantage of this approach is that the probe is capable of differentiating a given DNA topology without any bias associated with variable probe concentration and/or the affinity of the probe. Therefore, this opens up the possibility of studying the interaction of small molecules with G‐quadruplexes in live cells and in real time.

**DAOTA‐M2** contains a triangulenium core,^12^ which possesses a carbocationic charge that is delocalised over an extended aromatic system. As a result, the triangulenium core is favourably polarised for end‐on π–π stacking with G‐quadruplexes or intercalation between DNA base pairs and gives rise to a strongly emissive π–π* transition. In addition, substitution of the triangulenium core can be achieved modularly through a range of synthetic routes, thereby enabling systematic evaluation of structure–function relationships.^13^, ^14^, ^15^, ^16^, ^17^, ^18^ Yet, with the exception of **TOTA**,^19^, ^20^, ^21^ and more recently **DAOTA‐M2** (Scheme 1),^11^ these properties have not been explored in the context of fluorescent probes for nucleic acids (and more specifically selective for a given topology).

**Figure 1:**
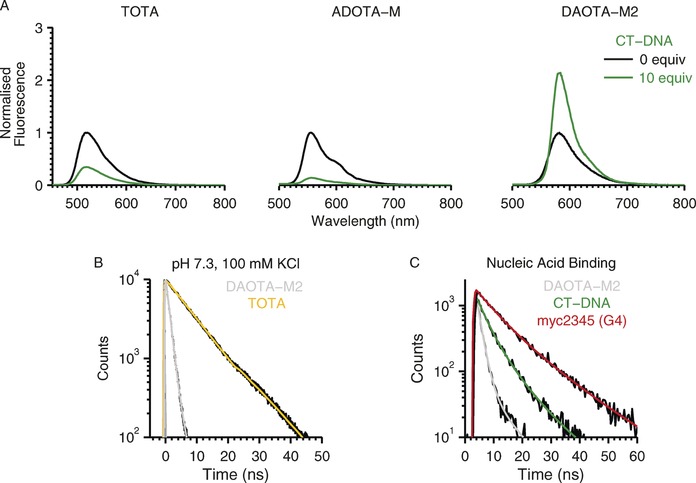
Unusual fluorescence lifetime properties of **DAOTA‐M2** depicts the effect of double stranded DNA (CT‐DNA, 10 base pair equivalents) on the emission of **TOTA**, **ADOTA‐M** and **DAOTA‐M2**. A) The spectra before (black) and after (green) addition of CT‐DNA (10 mm lithium cacodylate buffer, pH 7.3, 100 mm KCl) are normalised to compound only intensity maximum to highlight the fluorescence quenching and enhancement observed (the quantum yields of the free dyes under this conditions are: *Φ*=0.100 for **TOTA**; *Φ*=0.042 for **ADOTA‐M** ^11^; *Φ*=0.032 for **DAOTA‐M2** ^11^). B) Fluorescence lifetime decay of **TOTA** (yellow, *τ*_1_=8.6 ns) and **DAOTA‐M2** (grey, *τ*_1_/*f*_1_=1.3 ns/93 %, *τ*_2_/*f*_2_=3.7 ns/8 %) at physiological conditions (10 mm lithium cacodylate buffer, pH 7.3, 100 mm KCl), as was previously reported. C) Dependence of **DAOTA‐M2** fluorescence lifetime on the nucleic acid topology (duplex CT‐DNA in green and G‐quadruplex myc2345 in red).

Several unusual observations distinguish **DAOTA‐M2** from other available triangulenium fluorescent dyes (Scheme 1). Firstly, **DAOTA‐M2** displays an uncharacteristically short fluorescence lifetime at physiological pH and ionic conditions compared to the long‐lived decays of unsubstituted **TOTA** (Figure 1 B) or other previously reported alkyl‐derivative trianguleniums in organic solvents.^22^ Secondly, unlike structurally related **TOTA** and **ADOTA‐M**, fluorescence of**DAOTA‐M2** is enhanced and not quenched in the presence of nucleic acids (Figure 1 A). Finally, and most fascinatingly, **DAOTA‐M2** fluorescence lifetimes are significantly higher in the presence of G‐quadruplexes compared to double stranded DNA (Figure 1 C).

Herein, we set out to elucidate the origins of these unusual observations, using photophysical, electrochemical and computational studies. We demonstrate that the interplay between inter‐ and intra‐molecular photo‐induced electron transfer (PET) quenching is essential to the observed response of the compounds to nucleic acids. We then identify conformational changes that could give rise to intramolecular PET, before establishing the importance of the morpholino groups in generating the desired G‐quadruplex‐selective fluorescence response. In doing so, we aim to rationalise the response of **DAOTA‐M2** fluorescence to nucleic acids for the development of smarter and more selective G‐quadruplex probes.

## Results and Discussion

The fluorescence of small molecules can be quenched via non‐radiative pathways including Dexter energy transfer, exciplex formation, Förster resonance energy transfer (FRET) and photo‐induced electron transfer (PET). In the case of nucleic acid binders, intermolecular PET is the most common quenching mechanism due to the low oxidation potential of nucleobases and in particular guanine (1.3 V vs. normal hydrogen electrode (NHE)).^23^ However, for **ADOTA‐M** and **DAOTA‐M2**, possible additional intramolecular PET quenchers are present in the form of covalently attached tertiary amine groups. We hypothesise that a complex interplay between inter‐ and intra‐molecular quenching mechanisms may explain why some of the trianguleniums have their fluorescence quenched upon binding to nucleic acids (e.g., **TOTA** and **ADOTA‐M**), whereas others are enhanced (e.g., **DAOTA‐M2**). To evaluate this hypothesis, we have investigated the energetic feasibility of both mechanisms and identified the influence of the substituents on these properties.

The energetic driving force for PET can be predicted using the following equation, which represents the Gibbs energy of photo‐induced electron‐transfer processes:

(1) $$\Delta G=e\left[E\left(0^{+}/0\right)-E\left(R/R^{-}\right)\right]-\Delta G_{0,0}-e^{2}/ɛd$$

in which *E*(0^+^/0) is the oxidation potential of guanine (1.3 V vs. NHE),^23^ *E*(*R*/*R* ^−^) is the experimentally determined reduction potential, Δ*G*_0,0_ is the S_0_→S_1_ transition energy that is experimentally determined from (*λ*_abs,max_+*λ*_em,max_) of the compounds at pH 7.3, and *e* ^2^/*ɛd* is the one‐electron charge‐separation factor, which can be normally omitted due to its very low energetic contribution.^24^ Values of Δ*G* below zero indicate that quenching is thermodynamically favourable.

### Correlation between the electrochemical and photophysical properties of trianguleniums

**Figure 2:**
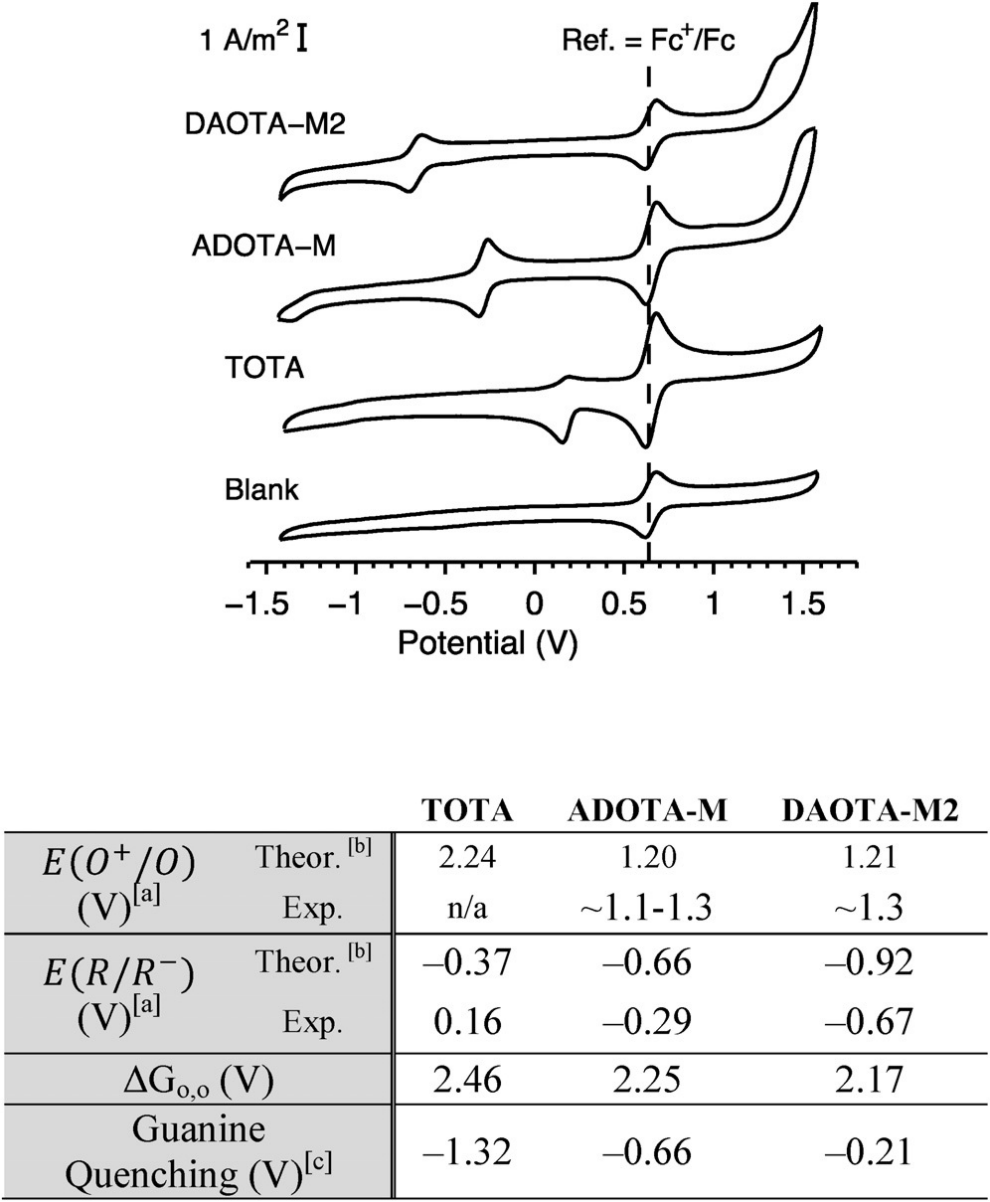
Electrochemical properties of **TOTA**, **ADOTA‐M** and **DAOTA‐M2**. Representative cyclic voltammograms were recorded in degassed acetonitrile containing 0.1 m *n*Bu_4_NPF_6_where ferrocene (Fc) is used as an internal standard (see the Supporting Information, Figure S1 for the scan‐rate dependence of various cyclic voltammogram parameters and Tables S1 and S2 for computational modelling energies used to determine theoretical electrochemical properties). [a] Versus NHE; [b] IEFPCM acetonitrile solvation; [c] Using the equation Δ*G*_0,0_=e[*E*(0^+^/0)−*E*(*R*/*R* ^−^)]−Δ*G*_0,0_−*e* ^2^/*ɛd*, in which *E*(0^+^/0) is the oxidation potential of guanine (1.3 V vs. NHE),^23^ *E*(*R*/*R* ^−^) is the experimentally determined reduction potential, Δ*G*_0,0_=(*λ*_abs,max_+*λ*_em,max_) at pH 7.3 and the negligible charge separation (*e* ^2^/*ɛd*) factor is omitted.^24^ Δ*G* values below zero indicate that quenching is thermodynamically favourable.

We first experimentally investigated the electrochemical properties of the trianguleniums **TOTA**, **ADOTA‐M** and **DAOTA‐M2** (Figure 2) using cyclic voltammetry. The values obtained were then supported theoretically using computational modelling (a DFT thermodynamic cycle approach; see Section 2.2 in the Supporting Information).

As shown in Figure 2, the substitution of oxo with aza bridges in the triangulenium core causes the first redox process associated with the reduction of the aromatic system to become progressively lower by approximately 0.4 V. This decrease is consistent with that predicted by theoretical calculations (ca. 0.3 V reduction in the series, see below). The linear dependence of peak potential on the square root of the scan rate suggests that the processes are diffusion controlled. At lower scan rates, single transitions that are stable with subsequent scans were observed. At higher scan rates, more complex transitions were also observed. The measured *i*_pa_/*i*_pc_ value remained constant, but was lower than one for all compounds. This suggests that the redox process may be more complex, perhaps involving ion pairing or coupled chemical processes. However, the mid‐point of the transitions remained constant with varying scan rate. Therefore, it was used to define an “effective” redox potential (see Figure S1 in the Supporting Information for scan‐rate‐dependent data). This assignment is supported by similar reduction potentials previously reported^25^ for *N*‐methyl **ADOTA** and **DAOTA‐Pr2** triangulenium derivatives (−0.26 and −0.61 V vs. NHE, respectively). Furthermore, these results suggest that the nature of substituent on the aza bridge does not significantly affect the redox potential of the fluorophore itself.

A second redox process was observed for **ADOTA‐M** and **DAOTA‐M2** in the region 1.1–1.3 V. This process is irreversible and most likely associated with amine‐based oxidation. Computational modelling of the **DAOTA‐M2** with aqueous solvation and protonation (see below) showed a shift in the oxidation potential only when both amines are protonated (ca. 0.4 V increase, see the Supporting Information). The geometry‐optimised oxidised forms of **ADOTA‐M** and **DAOTA‐M2** clearly indicate that in the oxidised state the tertiary amine adopts a trigonal planar arrangement consistent with localisation of a radical at the amine centre—an effect not seen if the amine is explicitly protonated (Figure S2 in the Supporting Information). Further evidence that this second redox process is associated to the amine group and not the aromatic core is provided by previous reports: *N*‐methyl **ADOTA‐M** displays no oxidation process around 1.1–1.3 V, and the oxidation process associated with the **DAOTA‐Pr2** aromatic core occurs at 1.64 V versus NHE.^25^

Based on these redox and excited state energy values, the values of the Gibbs energy of photoinduced electron‐transfer processes suggests that the fluorescence of all three trianguleniums should be quenched upon binding to nucleic acids (Δ*G*<0). Indeed, this correlates well with experimental observations for **TOTA** and **ADOTA‐M** (Figure 1). However, for **DAOTA‐M2**, fluorescence enhancement rather than quenching was observed upon binding to DNA. The key to understanding this observation is that the redox process associated with the morpholino group(s) is lower (1.1–1.3 vs. NHE) or at least equivalent to that of guanine (1.3 V vs. NHE). Therefore, intramolecular amine‐based PET quenching may supersede any intermolecular nucleic‐acid based PET quenching, and be alleviated upon binding to nucleic acids. As a result, overall, fluorescence enhancement was observed upon DNA binding. This rationale appears to support experimental observations for **DAOTA‐M2** as is discussed below.

**ADOTA‐M** represents an intermediate case: although it contains a mono‐morpholino substituent, its fluorescence is quenched in the presence of DNA (Figure 1). Thus, the redox properties alone fail to explain the photophysical behaviour of **ADOTA‐M**; its fluorescence lifetime decay at physiological pH must be considered to gain further understanding. These measurements, taken at the nanosecond timescale, showed that there is a significant proportion of long‐lived unquenched species, as well as short‐lived quenched species in solution of **ADOTA‐M** only (*τ*_1_/*f*_1_=0.4 ns/20 %, *τ*_2_/*f*_2_=18.3 ns/80 %). Yet, the p*K*_a_* of the compound is 4.7, thus, excluding the presence of protonated species.^11^ This data, taken together with the electrochemical studies, suggest that the overall quenching of **ADOTA‐M** fluorescence intensity in the presence of DNA is due to a delicate interplay of 1) partial intramolecular amine‐based PET; 2) DNA binding alleviating this effect; and 3) nucleobase intermolecular nucleobase‐based PET quenching.

It is interesting to note that fluorescence lifetime measurements for **DAOTA‐M2** displayed one major component when free in solution (*τ*_1_/*f*_1_=1.3 ns/93 %, *τ*_2_/*f*_2_=3.7 ns/8 %; Figure 1). This suggests that the presence of two morpholino groups is sufficient for the majority of the fluorescence to be quenched at neutral pH. This data also implies that the conformational change (that we believe is responsible for the reduced fluorescence lifetime of the dyes compared to that in unsubstituted **TOTA**) is related to the relative position of the triangulenium core and morpholino groups. The following section presents some structural information for **ADOTA‐M** to shed light on the relative position of the planar core and the morpholino substituent.

### X‐ray crystal structures of ADOTA‐M

**Figure 3:**
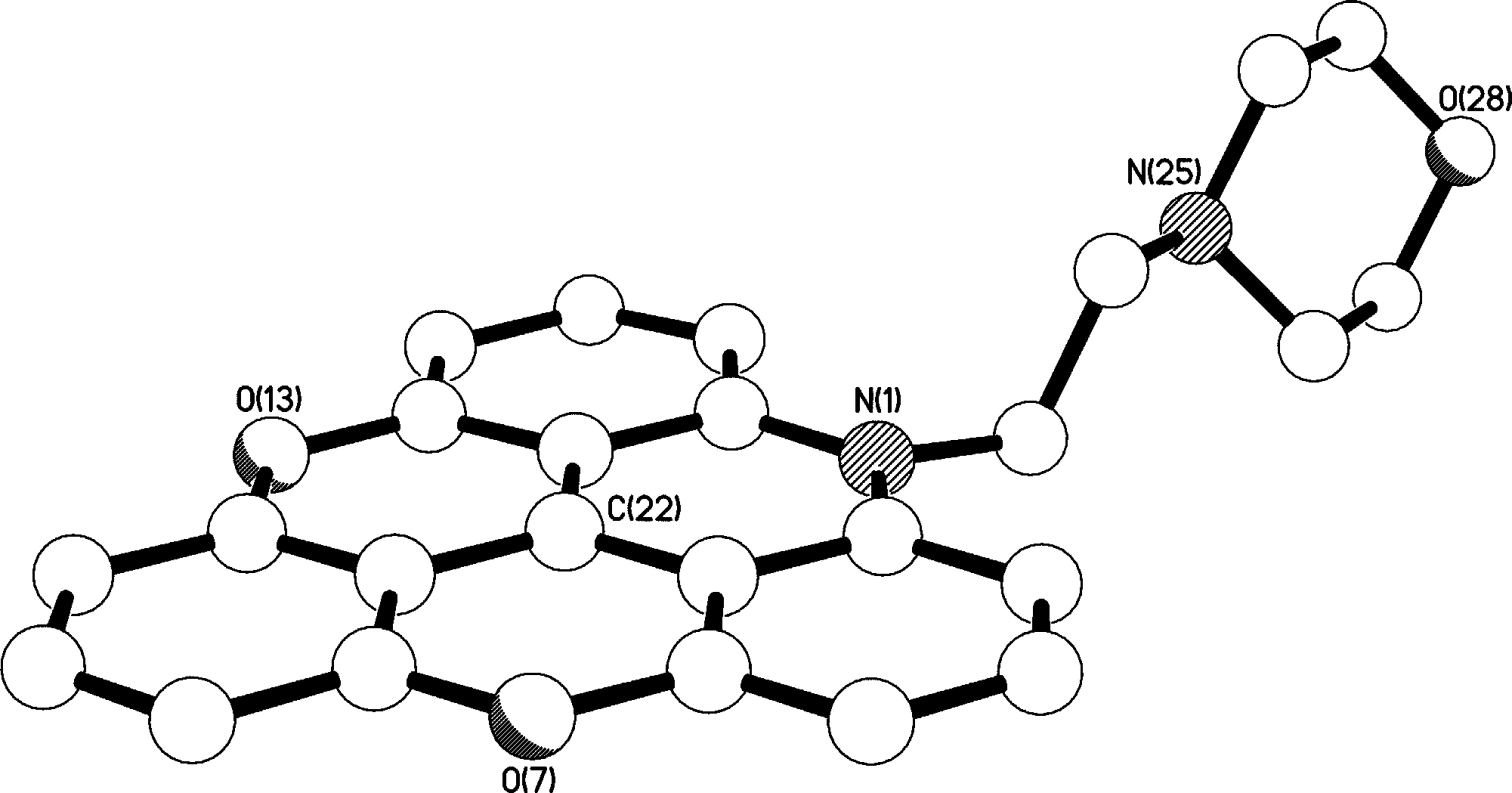
X‐ray crystal structure of the cation present in the crystals of **ADOTA‐M**.

**Figure 4:**
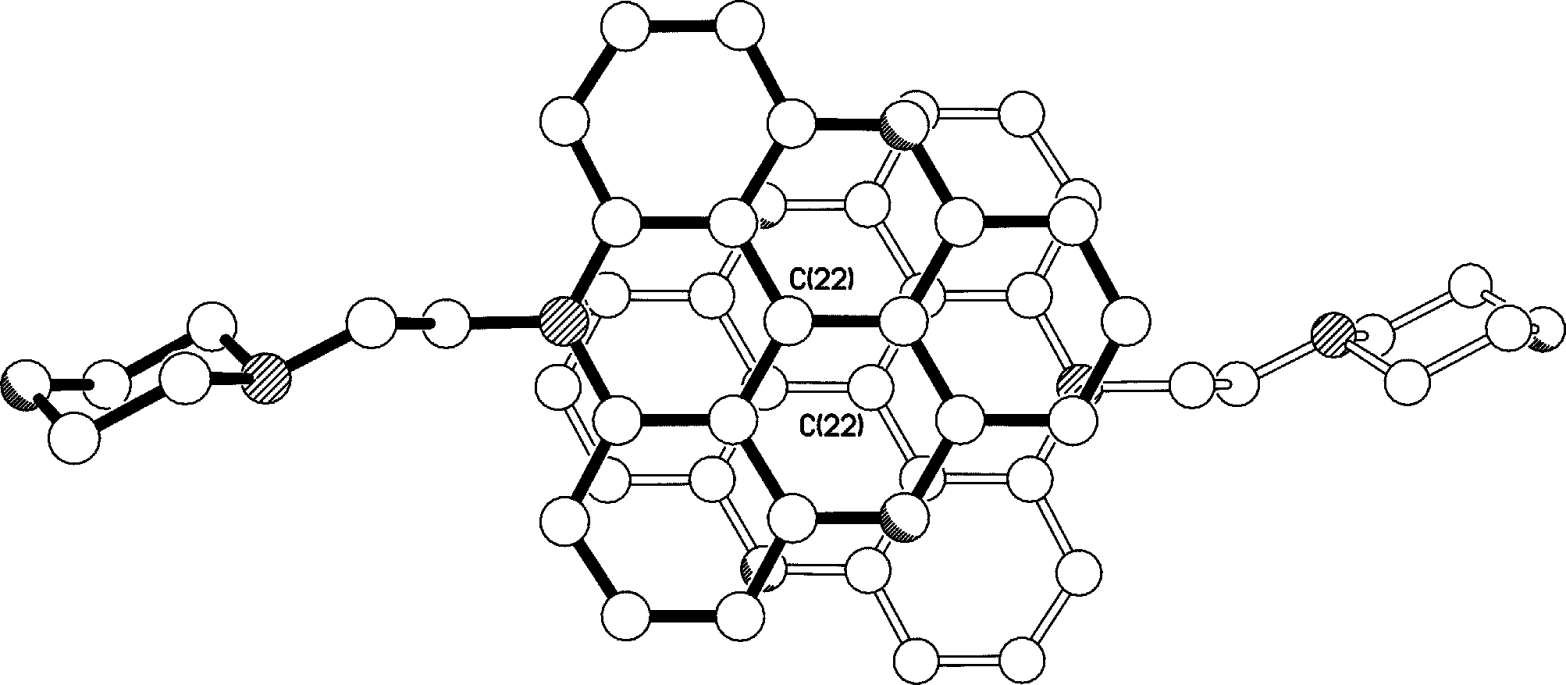
View in parallel projection perpendicular to the triangulenium ring plane, showing the head‐to‐tail overlap of the triangulenium units in adjacent, centro‐symmetrically related, cations in the structure of **ADOTA‐M**. The C22**⋅⋅⋅**C22 separation between the neighbouring cations is approximately 3.67 Å, with the mean interplanar separation being approximately 3.40 Å.

The triangulenium ring system in the structure of **ADOTA‐M** is essentially flat, the twenty two non‐hydrogen atoms being coplanar to within approximately 0.05 Å (Figure 3). The N‐CH_2_‐CH_2_‐N linkage between the triangulenium and morpholino ring systems is significantly twisted away from an ideal *anti* conformation, the torsion angle being 152.05(19)°; the associated N**⋅⋅⋅**N separation is 3.712(3) Å. Adjacent, centro‐symmetrically related, cations pack such that the triangulenium ring system of one ion substantially overlays the equivalent ring system of the other in a head‐to‐tail fashion (Figure 4); the separation between the “central” C22 atoms in the neighbouring cations is approximately 3.67 Å, with the mean interplanar separation being approximately 3.40 Å (the two ring systems are parallel as a consequence of the centre of symmetry).

### Computational DFT studies

**Figure 5:**
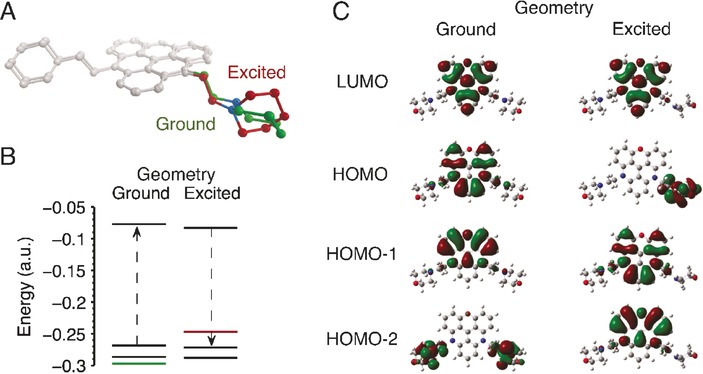
TD‐DFT studies of the effect of the excited (S_1_) and ground (S_0_) state geometries on the ordering of molecular orbitals in **DAOTA‐M2** and its impact upon emission. A) Superimposed optimised ground and excited state geometries. C−H bonds are omitted for clarity, and the main structural differences observed are highlighted in green (ground) and red (excited). B) Molecular orbital energies for both conformations. The dashed arrows indicate the first excited state transition as predicted by TD‐DFT (>90 % orbital contribution). C) HOMO−2 to LUMO ordering of the molecular orbitals for each geometry (see the Supporting Information, Figure S2 for **ADOTA‐M** data and Table S3 for oscillator strength, energies and configuration interaction (CI) coefficients).

Given that the conformations are likely to be influenced by nucleic acid binding, we conducted computational studies (using Gaussian 09^26^) to identify the conformational change involved in quenching. This computational data was supported by X‐ray crystal structures when possible. Time‐dependent (TD)‐DFT calculations were then performed to model excited state conformational changes and their effect upon electronic transitions in trianguleniums under study (Figure 5). Initially, the ground state (S_0_) geometries were optimised before being recalculated at the excited state (S_1_) by TD‐DFT using the same basis set and functionals.

In the ground state, the crystallographic and computationally modelled **ADOTA‐M** geometries are similar. The C(22)−C bond lengths are within 0.015 Å, whereas the C_alkyl_‐C‐*N*‐C_morph_ dihedral angle is twisted from 179.8° in the crystal structure to 159.0° in the DFT‐modelled structure. This difference is likely due to the short‐range contacts between the hexafluorophosphate anion and morpholino group in the crystal structure that are not present in the modelled structure (when the anion is omitted). This is supported by the same dihedral angle being 154.5° in the crystal structure of reduced **TMPA‐M** (i.e., when C−H bond is formed with the carbocation, see Figure S10 in the Supporting Information). In the case of **DAOTA‐M2**, the orientation of the morpholino groups with respect to the alkyl linkers was consistent with the DFT‐calculated **ADOTA‐M** structure (158.3°), whereas the relative orientation of two alkyl linkers, *syn* or *anti*, to the aromatic system had a negligible effect upon the overall energy of the molecule (0.08 kcal mol^−1^).

In the excited state, for both compounds, the hybridisation of the morpholino nitrogen changes from sp^3^ to sp^2^. Although the chair conformation is retained, the C_alkyl_‐C‐*N*‐C_morph_ dihedral angle increases to 175.4–176.9°. For **DAOTA‐M2**, this conformational change only occurs in one of the two morpholino groups. Furthermore, it should be noted that the addition of a diffuse function for more accurate modelling of the excited state did not alter the final geometries obtained. Interestingly, upon explicit protonation of the morpholino groups, there are no noticeable differences in geometry between the ground and excited states.

The effect of this conformational change on electronic transitions was then explored by TD‐DFT.^27^, ^28^ To more accurately describe long‐range electronic interactions, the basis set was expanded and the CAM‐B3LYP functional was used.^29^ Although in the ground‐state geometries, the absolute values of S_0_→S_1_ are different from experimentally observed values for both compounds, the errors are systematic (Δ*λ*_theor−exptl_≈105–114 nm). This implies that the qualitative descriptions provided by these calculations are reliable.

Theoretically, assuming Kasha's rule is valid, emission only occurs from the lowest excited state (S_1_). Thus, the S_0_→S_1_ excitation should be equivalent to S_1_→S_0_ transition in terms of the molecular orbitals involved. Furthermore, if the change in conformation upon excitation has no effect on emission, the ordering of the molecular orbitals should remain constant. However, for both **ADOTA‐M** and **DAOTA‐M2**, this is not the case. In the ground‐state geometry, the expected single electron π–π* HOMO→LUMO transition is observed. Yet, in the excited‐state geometry, the single‐electron π–π* transition corresponds to a HOMO−1→LUMO transition. The HOMO is instead localised on the morpholino group. It is possible to envisage that, upon excitation, the higher energy HOMO donates electron density to the partially occupied HOMO−1 in a n–π transition, thereby preventing the allowed emissive HOMO–LUMO π–π* transition; as a consequence, the excited state to the ground state through non‐radiative pathways and overall the observed fluorescence is quenched.

**Figure 6:**
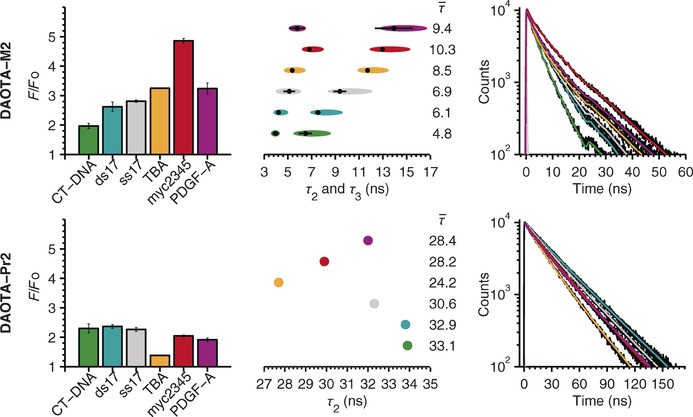
Top: **DAOTA‐M2** and bottom: **DAOTA‐Pr2** fluorescence enhancements (left), lifetimes (middle) and decay traces (right) in the presence of various DNA topologies (colour coded). The average lifetime ($\bar{\tau }$ /ns, *σ*=±5 %, *n*=3) and fluorescence enhancement were calculated from the end point of the corresponding DNA titrations. The decay traces shown are representative of the end point of titrations for the specific DNA topology. Error bars in the left graphs and horizontal black lines in the middle graphs indicate two standard deviations from three independent repeats. For the **DAOTA‐M^2^** lifetime graph (top middle), ellipses indicate accuracy of *τ*_2_ and *τ*_3_, was as determined by *F*_χ_ statistic (*F*_*χ*_=1.028/1.029, *P*=0.05, *p*=33/29, *v*=1600/1400 for CT‐DNA/other DNA models, respectively) from the systematic variation of *τ* over the lifetime range. For the **DAOTA‐Pr2** lifetime graph (bottom middle), parameter accuracy was not determined, because only one lifetime component was being determined in addition to the free dye. For complete DNA titration decay fittings, numerical *τ* and plots of the relative contributions of lifetime components at local *χ* ^2^_R_ minima, see the Supporting Information, Figures S6 and S7.

Intramolecular PET has often been rationalised in literature using the above‐mentioned arguments,^28^, ^30^ and appears pertinent in this case both experimentally and theoretically. It is important to note that, theoretically, this quenching process is only observed if the morpholino group can adopt the appropriate conformation. Hence, upon interaction with different nucleic acid topologies, it is likely that a complex range of conformations is adopted, thereby alleviating the intramolecular quenching to differing extents. This, in combination with other factors, such as through space effects,^31^ may explain the spread of **DAOTA‐M2** lifetimes observed in the presence of nucleic acids with binding to different topologies resulting in starkly different time resolved decays (Figure 6).

### Photophysical studies of the interaction of trianguleniums with DNA

To corroborate the proposed fluorescence mechanism, **DAOTA‐Pr2**, a previously reported *N*‐propyl triangulenium derivative that lacks the morpholino groups,^17^ was synthesised (Scheme 1). Its fluorescence response to interactions with various nucleic acid topologies was then compared to that of **DAOTA‐M2**. Initially, simple aqueous solvent models were considered, because fluorescence changes upon binding to biomolecules are usually a composite of local solvent environment factors. At physiological pH and ionic strength (10 mm lithium cacodylate buffer, pH 7.3, 100 mm KCl), the **DAOTA‐Pr2** fluorescence lifetime decay could be fitted using a mono‐exponential model (*τ*=15.8 ns), whereas **DAOTA‐M2**, as was previously reported, required a bi‐exponential fitting model with a very short major component (*τ*_1_/*f*_1_=1.3 ns/93 %, *τ*_2_/*f*_2_=3.7 ns/8 %, p*K*_a_*=4.5). Similar to **DAOTA‐M2**, **DAOTA‐Pr2** emission was independent of ionic strength (0–220 mm KCl) and degassing of the solution. However, unlike **DAOTA‐M2**, **DAOTA‐Pr2** emission was unresponsive to pH, and its fluorescence intensity and life‐time was enhanced—not reduced—in lower polarity 1,4‐dioxane/water solvent mixtures (1.5‐fold enhancement, 17.8 ns, see the Supporting Information, Figure S5). These results indicate that the short **DAOTA‐M2** lifetime is indeed due to intramolecular PET, and that this effect overrides any local solvent‐environment polarity‐based effects.

Upon the addition of nucleic acids, contrary to the quenching predicted by the Rehm–Weller equation, **DAOTA‐Pr2** emission was moderately enhanced (1.4–2.3‐fold). This is consistent with a response induced by a local environment with lower polarity/viscosity and suggests that this effect is stronger than nucleobase intermolecular PET quenching. Intriguingly, the enhancement in fluorescence intensity for the morpholino‐containing **DAOTA‐M2** is greater (2.0–4.9‐fold) and also more pronounced for G‐quadruplexes (TBA, myc2345, PDGF‐A) compared to double‐ (CT‐DNA, ds17) and single‐ (ss17) stranded DNA (Figure 6). Indeed, the different response of **DAOTA‐M2** and **DAOTA‐Pr2** to nucleic acid topologies is most evident when fluorescence lifetimes are considered (Figure 6).

We have recently reported **DAOTA‐M2** fluorescence lifetimes in the presence of nucleic acids, using a custom‐built‐time‐correlated single photo‐counting fluorescence lifetime imaging microscope (TCSPC‐FLIM).^11^ Herein, we used a standard single cuvette TCSPC setup to recover fluorescence lifetimes with higher accuracy and precision due to the higher peak counts achievable (*σ*=±5 %). In these experiments, DNA was titrated into a buffered solution containing the compound only and the fluorescence lifetime decay recorded after each addition. The multiple decay traces acquired per DNA model were then fitted using global analysis approach, that is, the lifetimes were “shared” for all decay traces, but the amplitudes were allowed to vary for each decay trace. Furthermore, to reduce parameter uncertainty, the unbound compound only lifetime was fixed to a value determined in a pure buffered solution: *τ*_1_=1.3 ns for **DAOTA‐M2** and *τ*_1_=15.8 ns for **DAOTA‐Pr2**. This methodology enabled parameter correlation to eliminate or reduce the influence of the fitted amplitudes on the lifetime values obtained.

For **DAOTA‐M2**, the lifetimes determined are identical to those reported previously by using TCSPC‐FLIM.^11^ In addition to the lifetime characteristic of free unbound species, two lifetime components were required to describe the fluorescence decay curves in the presence of nucleic acid binding. Uniquely, the longer of the two components offers clear discrimination between G‐quadruplexes and double‐/single‐stranded DNA (Figure 6). In contrast, for **DAOTA‐Pr2**, only one additional lifetime component was required. Moreover, the selectivity for the lifetime response of the two dyes is inverted, namely, **DAOTA‐Pr2** displays shorter lifetimes in the presence of G‐quadruplexes compared to those in the presence of double‐/single‐ stranded DNA (Figure 6).

**Figure 7:**
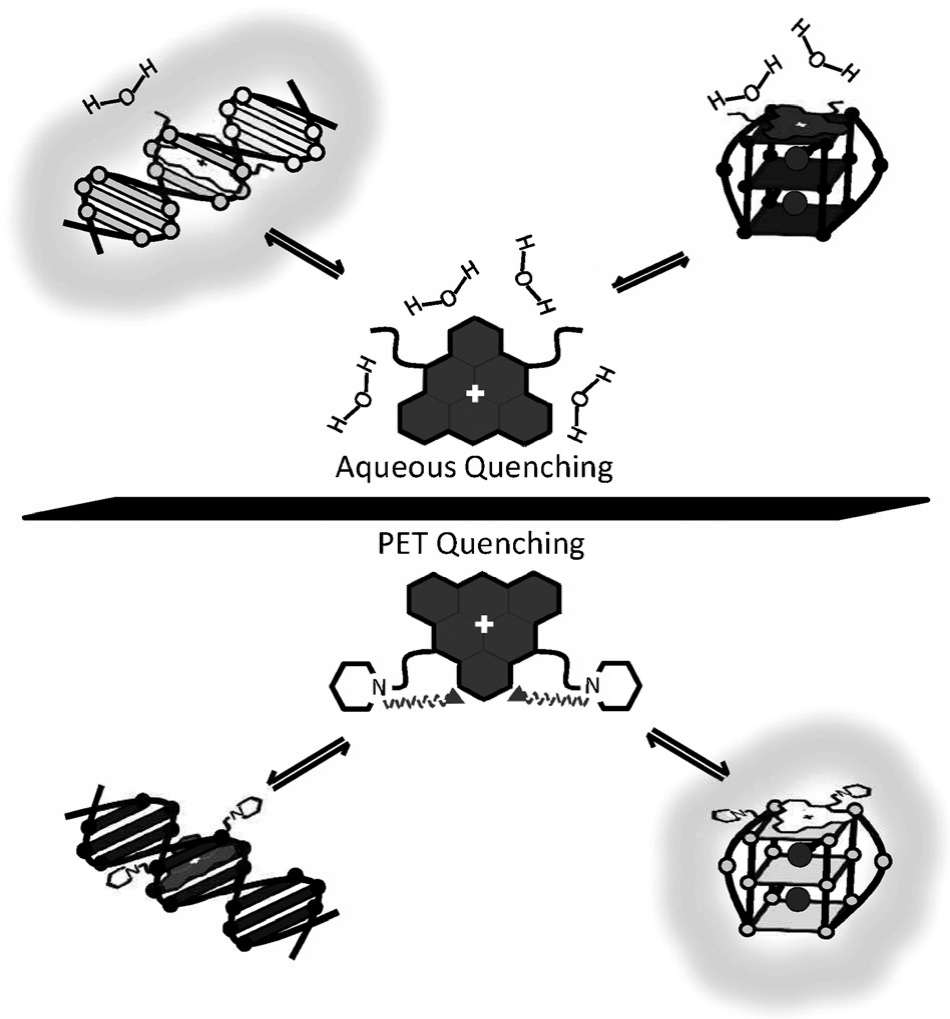
Schematic representation of the proposed fluorescence response of **DAOTA‐Pr2** (top) and **DAOTA‐M2** (bottom) to double stranded DNA (left) and G‐quadruplexes (right). **DAOTA‐Pr2** fluorescence is quenched by exposure to the local aqueous solvent environment. This effect is alleviated to a greater extent upon intercalation between base pairs in duplex DNA as opposed to end‐on π–π stacking with G‐quadruplexes. **DAOTA‐M2** fluorescence is predominately controlled by intramolecular photo‐induced electron transfer (PET). This conformation‐dependent quenching process is alleviated to a greater extent upon binding G‐quadruplexes, opposed to duplex DNA.

These observations clearly demonstrate that the presence and conformations of morpholino groups alter the fluorescence response of the triangulenium core in the presence of different nucleic acid topologies (Figure 6). As was noted previously, **DAOTA‐Pr2** emission appears to be mainly controlled by local solvent environment polarity/viscosity. Therefore, the shorter lifetimes that were observed in the presence of G‐quadruplexes indicates that the binding site is more exposed to the local aqueous solvent environment than for double stranded DNA (Figure 7). Given the large aromatic system of the compound, this is consistent with external end‐on π–π stacking with G‐tetrads compared to intercalation between double‐stranded DNA base pairs. Conversely, **DAOTA‐M2** emission is predominately controlled by the intramolecular PET quenching effect of the morpholino groups. The longer lifetimes observed in the presence of G‐quadruplexes suggest that these groups are more engaged upon binding to G‐quadruplexes than to double‐stranded DNA, and therefore, less able to quench the triangulenium fluorescence. Furthermore, the fact that two components are required to describe **DAOTA‐M2** binding implies that, either the morpholino groups give rise to non‐specific binding, or that there are sub‐populations of the compound that are subject to less quenching than others at the timescale of fluorescence. The former scenario is unlikely given the large proportions of each lifetime component (*τ*_2_ *f*≈30–40 %; *τ*_3_ *f*≈45–65 %). The latter scenario, on the other hand, is supported by the conformational dependence of intramolecular PET discussed previously and the fact that **DAOTA‐M2** contains two morpholino groups.

Finally, it is interesting to note that the excited state affinity constants for **DAOTA‐Pr2** are very similar to those of **DAOTA‐M2** (Table 1). The main difference lies in double‐stranded DNA binding constants, in which **DAOTA‐M2** affinity is approximately five times lower. This suggests that the factors that give rise to fluorescence selectivity do not cause significantly stronger binding interactions. It is also noteworthy that the affinity constants of **DAOTA‐M2** to various DNA topologies are not vastly different, whereas the fluorescence lifetimes still show stark and characteristic changes upon binding to G‐quadruplexes versus duplex DNA. This observation gives us confidence that while a useful discrimination between binding to duplex and quadruplex DNA can be obtained, the presence of the probe is not likely to “force” the DNA into a certain topology, due to an extremely high binding affinity. We believe that this factor favourably distinguishes our new G‐quadruplex sensor from other fluorescent G‐quadruplex probes reported in the literature.^9^, ^10^

**Table 1:** **DAOTA‐Pr2** affinity constants for G‐quadruplexes (not bold) and double‐/single‐stranded DNA (bold), as was determined by emission titrations.

*K*_a_ [m ^−1^]	**DAOTA‐M2** ^[a]^	**DAOTA‐Pr2**
**CT‐DNA**	**7.9±0.8×10^5^**	**8.1±3.5×10^6^**
**ds17**	**4.5±0.7×10^5^**	**2.1±0.3×10^6^**
**ss17**	**5.3±1.5×10^5^**	**1.0±0.2×10^6^**
		
TBA	1.0±0.2×10^6^	1.2±0.4×10^6^
myc2345	1.2±0.5×10^6^	8.4±2.9×10^5^
PDGF‐A	7.6±2.7×10^5^	1.6±1.5×10^6^

[a] **DAOTA‐M2** affinity constants were reported previously^11^ and are reproduced herein. Errors displayed represent the standard deviation of three independent repeats. For binding isotherms, stoichiometry and numerical error bars, see the Supporting Information, Figure S8.

## Conclusion

Taken together, these results demonstrate that nucleobases can quench the fluorescence of triangulenium derivatives by intermolecular PET. However, this process becomes energetically unfavourable with the introduction of each aza bridge into the triangulenium structure. As a result, for bis‐substituted **DAOTA** derivatives, other factors, such as local environment effects (**DAOTA‐Pr2**) can override PET quenching with nucleotides. Usefully, the compound's fluorescence response to different nucleic acid topologies could be further altered by the addition of covalently attached tertiary amine groups (**DAOTA‐M2**). These groups give rise to intramolecular PET quenching—an effect that supersedes intermolecular nucleobase PET quenching and local solvent polarity/viscosity environment effects. Consequently, we found that this quenching effect is alleviated to differing degrees upon binding to nucleic acids and is strongly topology dependent. The structural features of triangulenium chromophores discussed herein highlight the fact that just as binding affinity and selectivity can be fine‐tuned for a target, so can the fluorescence response. In principle, the intramolecular PET quenching mechanism demonstrated herein should work with other nucleic acid binding fluorophores and may generate fascinating responses in probes designed for recognising non‐canonical nucleic acid topologies.

## Experimental Section

### General procedures

All chemicals were used as purchased from commercial sources unless stated otherwise. Solvents were distilled over an appropriate drying agent and degassed prior to use. ^1^H and ^13^C NMR spectra were recorded by using a 400 or 500 mhz Bruker Avance Ultrashield NMR spectrometer at 296 K, respectively. Chemical shifts are referenced to residual deuterated solvent. Mass spectra were obtained by using electrospray ionisation (ESI) by Mrs. L. Haigh (Imperial College London) on a Bruker Daltronics Esquire 3000 spectrometer. Compound microanalysis was performed by Mr. A. Dickerson (Cambridge University). Absorption measurements were made on a Perkin–Elmer UV/Vis spectrometer. Emission spectra were obtained on a Varian Cary‐Eclipse fluorescence spectrometer. The oligonucleotides used were purchased RP‐cartridge purified from Eurogentec. CT‐DNA was obtained from Sigma–Aldrich. **TOTA**, **TMPA‐M**, **ADOTA‐M**,**DAOTA‐M2**,^11^ and 1,13‐dimethoxy‐5,9‐bis (propyl)‐5*H*‐quinolino[2,3,4‐kl]acridin‐9‐ium tetraflouroborate^13^ were prepared by previously reported procedures.

### Synthesis of 8,12‐bis(*n*‐propyl)‐8*H*‐benzo[ij]xantheno[1,9,8‐cdef][2,7]naphthyridin‐12‐ium hexafluorophosphate (DAOTA‐Pr2)

This compound was prepared using a slightly modified procedure to that reported by Laursen et al.^17^ Pyridine hydrogen chloride (2.50 g) was mixed thoroughly with 1,13‐dimethoxy‐5,9‐bis(propyl)‐5*H*‐quinolino[2,3,4‐kl]acridin‐9‐ium tetraflouroborate (0.07 g, 0.14 mmol) and dried under vacuum before the solid was stirred and heated to 200 °C for 1 h, shielding from light. The deep red solution was cooled to room temperature, and water (5 mL) was added. To the red suspension, an aqueous solution of ammonium hexafluorophosphate (0.2 m, 10 mL) was added, and the mixture was stirred for 10 min. The magenta precipitate was collected by filtration, washed with water (5×15 mL), and dried. Repeated crystallisation from a mixture of methanol and acetonitrile afforded red block crystals of **DAOTA‐Pr2**. Yield: 18 mg, 0.04 mmol, 27 %. ^1^H NMR (400 mhz, [D_6_]DMSO): *δ*=8.23 (t, 1 H, ^3^J_HH_=8.7 Hz,ArH‐10), 8.05(dd,2 H, ^3^ *J*_HH_=9.2 and 8.2 Hz, ArH‐2 and H‐6), 7.66 (d, 2 H, ^3^ *J*_HH_=9.2 Hz, ArH‐1 and H‐7), 7.56 (d, 2 H, ^3^ *J*_HH_=8.7 Hz, ArH‐9 an H‐11), 7.27 (d, 2 H, ^3^ *J*_HH_=8.2 Hz, Ar H‐3 and H‐5), 4.49–4.37 (m, 4 H, NCH_2_), 1.87–1.73 (m, 4 H, NCH_2_CH_2_), 1.12 ppm (t, 6 H, ^3^ *J*_HH_=7.2 Hz, CH_3_); ^13^C NMR (101 mhz, [D_6_]DMSO): *δ*=151.8, 140.3, 139.5, 139.2, 138.4, 111.1, 109.4, 108.1, 107.1, 105.9, 48.4, 18.8, 10.7 ppm; ^19^F NMR (377 mhz, [D_6_]DMSO): *δ*=69.7 (d, ^1^ *J*_PF_=710.1 Hz, PF^−^_6_), −148.2 (BF^−^_4_); MS (ES^+^) *m*/*z* 367.2 [*M* ^+^−(BF_4_)_0.75_(PF_6_)_0.25_], in which *M*=C_25_H_23_N_4_O(BF_4_)_0.75_(PF_6_)_0.25_); *Φ*=0.27 (in 10 mm lithium cacodylate buffer, pH 7.3, 100 mm KCl); elemental analysis calcd (%) for C_25_H_23_N_4_O(BF_4_)_0.75_(PF_6_)_0.25_ (*M*_w_=468.67): C 64.05, H 4.95, N 5.98 %; found C 64.37, H 5.02, N 5.76.

### Crystal data for ADOTA‐M

[C_25_H_21_N_2_O_3_][PF_6_], *M*=542.41, monoclinic, *P*2_1_/*c* (no. 14), *a*=8.99082(11), *b*=12.25787(15), *c*=20.4547(3) Å, *β*=98.7567(12)°, *V*=2228.00(5) Å^3^, *Z*=4, *ρ*_calcd_=1.617 g cm^−3^, *μ*Cu_Kα_=1.865 mm^−1^, *T*=173 K, orange red shards, 4349 independent measured reflections (*R*_int_=0.0304), *F* ^2^ refinement,^32^, ^33^ *R*_1_(obs)=0.0563, *wR*_2_(all)=0.1717, 3851 independent observed absorption‐corrected reflections [|*F*_o_|>4*σ*(|*F*_o_|), 2*θ*_max_=145°], 393 parameters. Recorded on Oxford Diffraction Xcalibur PX Ultra diffractometer. CCDC  963957 contains the supplementary crystallographic data for this paper. These data are provided free of charge by The Cambridge Crystallographic Data Centre.

### Cyclic voltammetry

Cyclic voltammograms were recorded in acetonitrile containing 0.1 m tetreabutylammonium hexafluorophosphate under an atmosphere of argon. The working electrode was a glassy carbon disc (diameter=2.5 mm), whilst reference and counter electrodes were platinum wires, respectively. Potentials are reported relative to the potential of the Fc^+^/Fc couple with respect to the normal hydrogen electrode (+0.64 V). Compound and reference concentrations were 0.1 mm. Three independent repeats were performed.

### Computational modelling

Ground‐state, one‐electron‐reduced and one‐electron‐oxidised geometries were optimised in the gas phase using the B3LYP/6–31G(d,p) level of theory. Geometries were compared, when possible, with crystal structure data. Vibrational frequency analysis confirmed convergence to local minima and enabled unscaled zero‐point energy and entropy corrects at room temperature to be calculated. Single‐point energies of the optimised structures were calculated by using the B3LYP/cc‐pVTZ level of theory in the gas phase and acetonitrile solvation using the polarisable continuum model using the integral equation formalism variant (IEFPCM). Geometries were not re‐optimised upon applying the solvation model. Oxidation and reduction potentials (vs. NHE, 4.4 V) were calculated by using the thermodynamic cycle.^34^ The specific equations used are:

(2) $$E\left(0^{+}/0\right)=-\frac{\Delta G_{\mathrm{gas},\mathrm{EA}}-\Delta G_{\mathrm{solv},0}+\Delta G_{\mathrm{solv},0}}{nF}$$

(3) $$E\left(R/R^{-}\right)=-\frac{\Delta G_{\mathrm{gas},\mathrm{EA}}-\Delta G_{\mathrm{solv},R}+\Delta G_{\mathrm{solv}{,R}^{-}}}{nF}$$

Excited‐state (S_1_) geometries were optimised by using TD‐DFT calculations using the B3LYP/6–31G(d,p) level of theory. To increase the reliability of the optimisation, the maximum allowed step size was reduced to 0.1 Bohr. When changes in geometry were observed, calculations were repeated by using a diffuse function to ensure the excited state is accurately described. Excitation energies, oscillator strength and the associated transitions were calculated by using the excited‐ and ground‐state geometries by using the CAM‐B3LYP/6–311G+(2d,p) level of theory and IEFPCM aqueous solvation.

### Aqueous solvent effects

**DAOTA‐Pr2** (2 μm) was excited at *λ*=404 nm when absorbance is minimal, and emission was monitored from 500 to 750 nm. Fluorescence enhancement or quenching was determined by the integrated fluorescence of the emission spectrum. pH 1.0 and 7.3 measurements were recorded in 0.1 m HCl and 10 mm lithium cacodylate buffer, containing 100 mm KCl respectively. Measurements in 1,4‐dioxane were recorded at pH 7.3. Ionic strength measurements were performed in 10 mm lithium cacodylate buffer (pH 7.3) by increasing KCl concentration from 0 to 220 mm.

### DNA annealing

The oligonucleotides used in this study are ds17 (equimolar 5′‐CCA‐GTT‐CGT‐AGT‐AAC‐CC‐3’ and 5′‐GGG‐TTA‐CTA‐CGA‐ACT‐GG‐3′), ss17 (5′‐CCA‐GTT‐CGT‐AGT‐AAC‐CC‐3′), TBA (5′‐GGT‐TGG‐TGT‐GGT‐TGG‐3′), myc2345 (5′‐TGA‐GGG‐TGG‐GGA‐GGG‐TGG‐GGA‐A‐3′) and PDGF‐A (5′‐GGA‐GGC‐GGG‐GGG‐GGG‐GGG‐GCG‐GGG‐GCG‐GGG‐GCG‐GGG‐GAG‐GGG‐CGC‐GGC‐3′). DNA was dissolved in 10 mm lithium cacodylate buffer (pH 7.3) containing 100 mm potassium chloride and annealed at 95 °C for 5 min before cooling to room temperature overnight. The concentration was checked by using the molar extinction coefficients 6600, 8910, 143300, 229900 and 467400 mol dm^−3^ cm^−1^, respectively. Annealing concentrations were approximately 5 mm for ds17 and ss17, whilst 1 mm for TBA and myc2345. For PDGF‐A, as was reported previously,^35^ the DNA was annealed in the same buffer but containing 25 mm potassium chloride at 15 μm concentrations to enable intramolecular folding.

### Time‐correlated single‐photon counting

Time‐resolved fluorescence decay traces were obtained by using a TCSPC JobinYvon IBH data station (5000F, HORIBA Scientific Ltd.) by using a 404 nm excitation source (200 ps pulse duration, HORIBA). **DAOTA‐M2** and **DAOTA‐Pr2** decays were recorded until at least 10 000 counts were collected in the peak maxima at an emission wavelength of 575±16 nm using an emission monochromator. Additionally, two long‐pass filters (>570 nm) were used in the detection channel to avoid light scattering reaching the detector. The instrument response function (IRF) measurements were performed using a Ludox solution, (using detection at 400±3 nm) using a neutral density filter (ND=2) in the detection channel. Three independent repeats of all time‐resolved fluoresce measurements were performed. Traces were fitted by iterative reconvolution to the equation *I*(*t*)=*I*_0_((1−*α*_1_−*α*_2_)*e* ^−*t*/*τ*1^+*α*_1_ *e* ^−*t*/*τ*2^+*α*_2_ *e* ^−*t*/*τ*3^, in which *α*_1_ and *α*_2_ are variables and the Σ*α* is normalised to unity. The fractional contribution to the steady state emission is calculated from the equation *f_i_*= *α*_1_ *τ_i_*/Σ_*j*_ *α_j_τ_j_*. The average lifetime was calculated using the equation $\bar{\tau }$ =Σ_*j*_ *α*_1*j*_ *τ_j_*. In the case of mono‐ or bi‐exponential decays, the *α*_2_ and/or *α*_3_ terms were set to zero. A “prompt shift” parameter was included in the fitting to take into account differences in the emission wavelength between the IRF and decay, as well as the different number of filters used. The goodness of fit was judged by consideration of the deviations from the model. Least‐square minimisation was performed by using the Quasi‐Newton algorithm in MatLab.^36^ For **DAOTA‐Pr2**, the lifetime channel spacing was similar to that of the IRF (220 ps). Therefore, the goodness of fit was determined after the peak of the IRF.

For DNA titrations, oligonucleotides were annealed as described in the section “DNA Annealing”. Compound concentration was held constant (2 μm, 10 mm lithium cacodylate buffer, pH 7.3, 100 mm potassium chloride) and concentrated DNA was then added to this solution in aliquots. PDGF emission titrations were performed by the addition of PDGF‐A (15 μm) containing **DAOTA‐M2**or **DAOTA‐Pr2** (2 μm) to a compound‐only solution. **DAOTA‐M2**/**DAOTA‐Pr2** was added to the PDGF‐A DNA to correct for dilution of **DAOTA‐M2**/**DAOTA‐Pr2** upon DNA titration. For **DAOTA‐M2**, the time‐resolved fluorescence decays were fitted to a tri‐exponential decay model using global analysis (i.e., the same *τ*_1_, *τ*_2_ and *τ*_3_ for all decay traces but variable *α*_2_ and *α*_3_ for each individual decay trace). To reduce parameter uncertainty, the unbound compound lifetime was held constant at 1.28 ns based on compound‐only measurements. Parameter precision, in the form of standard deviation, was determined by allowing all parameters except the compound only lifetime to minimise to a global minimum for three independent repeats. Accuracy was determined by holding one of the two free *τ* at a constant value in 1 ns intervals from 2 to 17 ns (with the exception of CT‐DNA, 0.5 ns intervals, 2–12.5 ns) and allowing the other *τ* and all other parameters to optimise. Value of *τ*_2_ is referred to as the short lifetime component and *τ*_3_ the longer component. The lowest global *χ*_r_^2^(parm) for a given *τ* value from three repeats was compared to the global minimum *χ*_r_^2^(min) when the fixed *τ* was also allowed to vary for the specific repeat in question. This was done by using the critical *F*_*χ*_ value (*F*_*χ*_=1.028/1.029, *P*=0.05, *p*=33/29, *v*=1600/1400 for CT‐DNA, other DNA models, respectively) calculated from the equation *F*_*χ*_=*χ*_r_^2^(parm)/*χ*_r_^2^(min)=1+(*p*/*v*)*F*(*P*,*v*,*p*). If the *F*_*χ*_ is below the critical value, all *τ* values are accepted. This creates a surface, in which local minima correspond to different *τ* values and their respective accuracy. The degrees of freedom for each trace (*v*) was set to 200 based on the generally accepted *χ*_r_^2^ for an acceptable time correlated single photon counting model of below 1.17 (*P*=0.05, *v*=200).^24^ For **DAOTA‐Pr2** DNA titrations, the time‐resolved fluorescence decays were fitted to a bi‐exponential decay model using global analysis. To reduce parameter uncertainty, the unbound compound lifetime was held constant at 15.75 ns based on compound only measurements. Given the reduced number of parameters, parameter precision was not determined.

### Emission titrations

**DAOTA‐Pr2** DNA titrations were performed as described in the Section “Time‐correlated single‐photon counting”. Fold enhancement of emission was determined by the fluorescence at the end of the titration divided by the initial fluorescence of the compound‐only spectrum. Samples were excited at 465 nm, and emission spectra were recorded from *λ*=500–800 nm. Three independent repeats were performed. Titrations curves were fitted by using 1:1 binding model using the emission values from *λ*=584 to 634 nm using a modified form of the MatLab (R2013a) script reported previously.^37^ The trust‐region‐reflective minimisation algorithm was used. The sudden change in gradient of the emission binding curves was used to determine the stoichiometry of binding (mole ratio method).^38^ This was a ratio of two compounds per five base pairs (CT‐DNA and ds17), one to five bases for ss17, one‐to‐one G‐quadruplex (TBA and PDGF‐A) and two compounds to one G‐quadruplex (myc2345). Association constants were then determined relative to the DNA‐binding motif, as was defined by the stoichiometry. For example, in the case of ss17, the binding constant is reported relative to a binding of one compound to a motif of five bases as opposed to one base. This is based on the assumption that there is no co‐operativity in binding, and enables all data to be modelled by a one‐to‐one compound to DNA‐binding‐motif stoichiometry.^39^

## Supporting information

As a service to our authors and readers, this journal provides supporting information supplied by the authors. Such materials are peer reviewed and may be re‐organized for online delivery, but are not copy‐edited or typeset. Technical support issues arising from supporting information (other than missing files) should be addressed to the authors.

Supplementary
