# Supplementary material for: Trianguleniums as Optical Probes for G‐Quadruplexes: A Photophysical, Electrochemical, and Computational Study
Source: Chemistry. 2016 Feb 16;22(12):4129–39. doi: 10.1002/chem.201504099 (PMC4991273; doi:10.1002/chem.201504099)
Supplement: Supplementary file 1 — Supplementary [file CHEM-22-4129-s001.pdf]

CHEMISTRY
A European Journal

Supporting Information

Trianguleniums as Optical Probes for G-Quadruplexes: A Photophysical, Electrochemical, and Computational Study

Arun Shivalingam, Aurimas Vyšniauskas, Tim Albrecht, Andrew J. P. White,
Marina K. Kuimova,* and Ramon Vilar*[a]

chem_201504099_sm_miscellaneous_information.pdf

TOTA

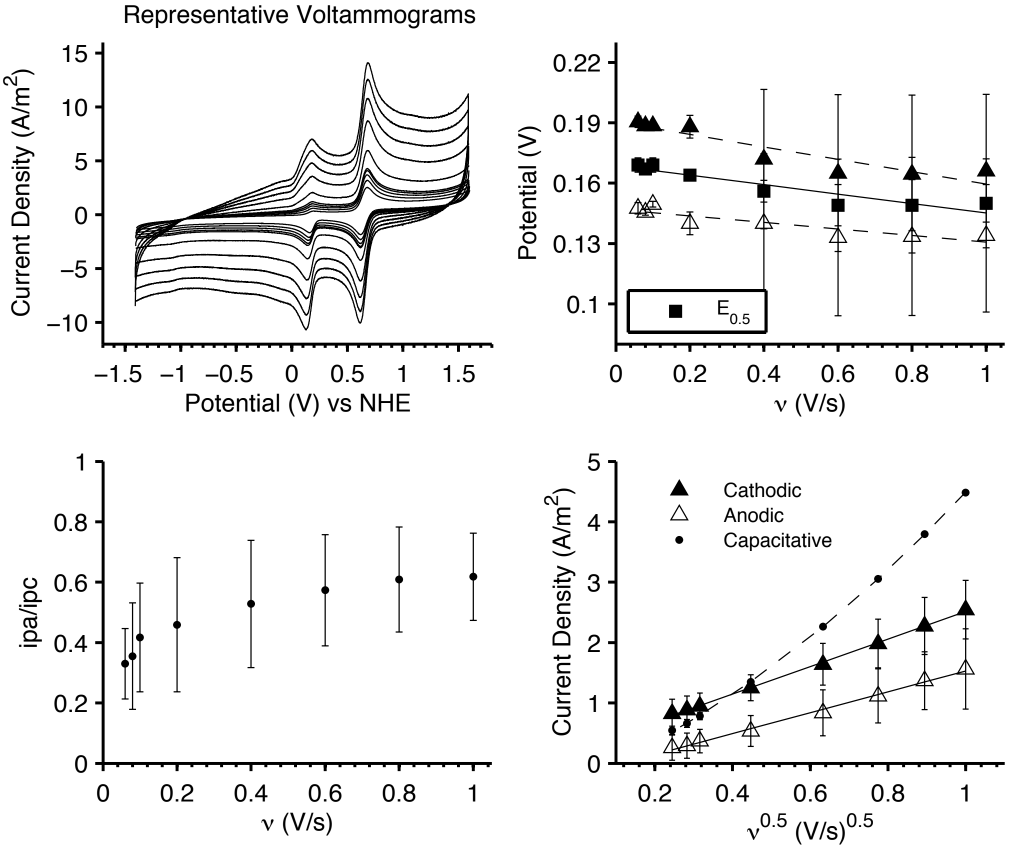
Representative Voltammograms
Current Density (A/m2)
Potential (V) vs NHE
Potential (V)
ν (V/s)
E0.5
ipa/ipc
ν (V/s)
Cathodic
Anodic
Capacitative
Current Density (A/m2)
ν0.5 (V/s)0.5

ADOTA-M

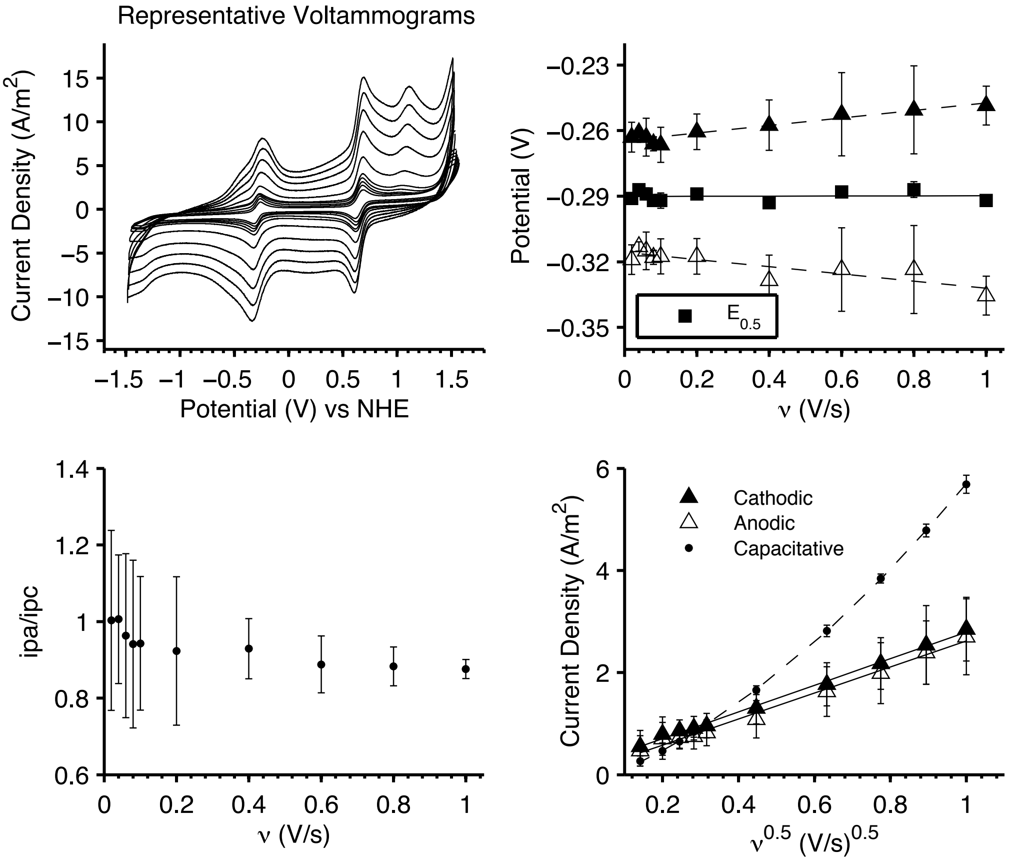
Representative Voltammograms
Current Density (A/m2)
Potential (V) vs NHE
Potential (V)
ν (V/s)
E0.5
ipa/ipc
ν (V/s)
Cathodic
Anodic
Capacitative
Current Density (A/m2)
ν0.5 (V/s)0.5

DOATA-M2

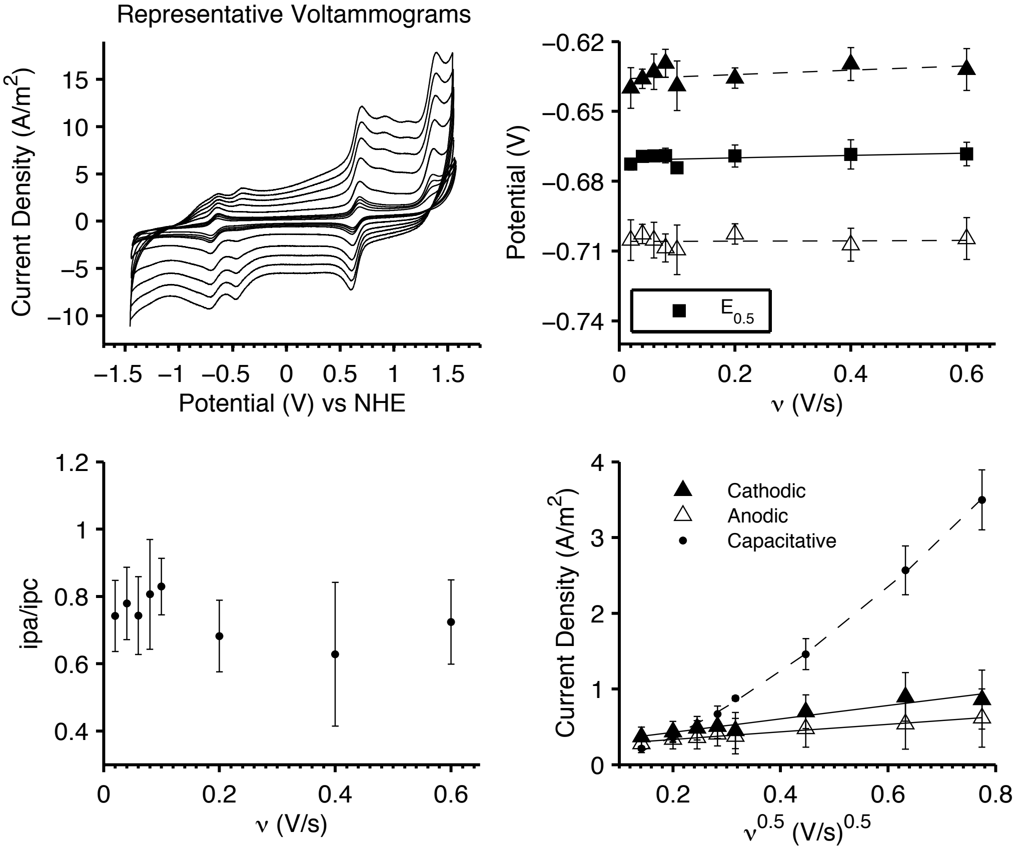

Figure S1. Representative **TOTA**, **ADOTA-M** and **DAOTA-M2** cyclic voltamograms and the scan rate dependence of various parameters (acetonitrile, 0.1 M tetrabutylammonium hexafluorophosphate, argon atmosphere). For **DAOTA-M2**, scan rate dependent parameters are for the transition that is retained at slow scan rates.

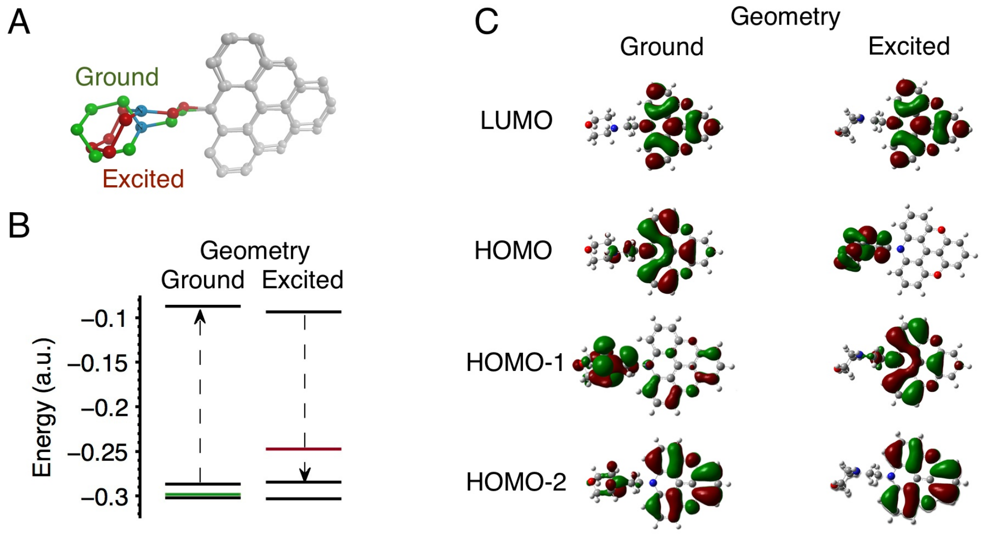

Figure S2. Time dependent DFT studies of the effect of the excited (S_1) and ground (S_0) state geometries on the ordering of molecular orbitals in **ADOTA-M** and its impact upon emission. (A) shows the superimposed optimised ground and excited state geometries. C−H bonds are omitted for clarity and the main structural differences observed are highlighted in green (ground) and red (excited). Image (B) shows the molecular orbital energies for both conformations. The dashed arrows indicate the first excited state transition as predicted by time dependent DFT (>90% orbital contribution). (C) shows the HOMO-2 to LUMO ordering of the molecular orbitals for each geometry.

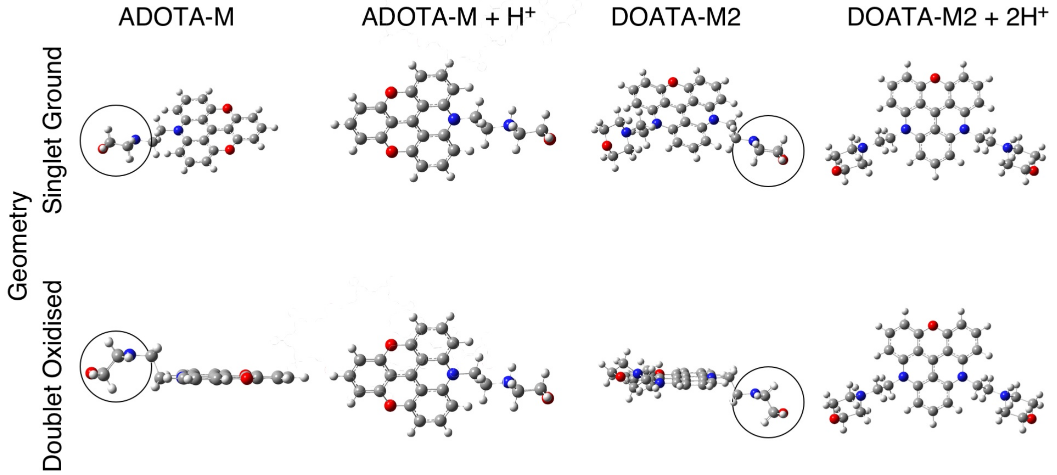

Figure S3. Thermodynamic cycle analysis optimised structures at the B3LYP/6-31G(d,p) level of theory.

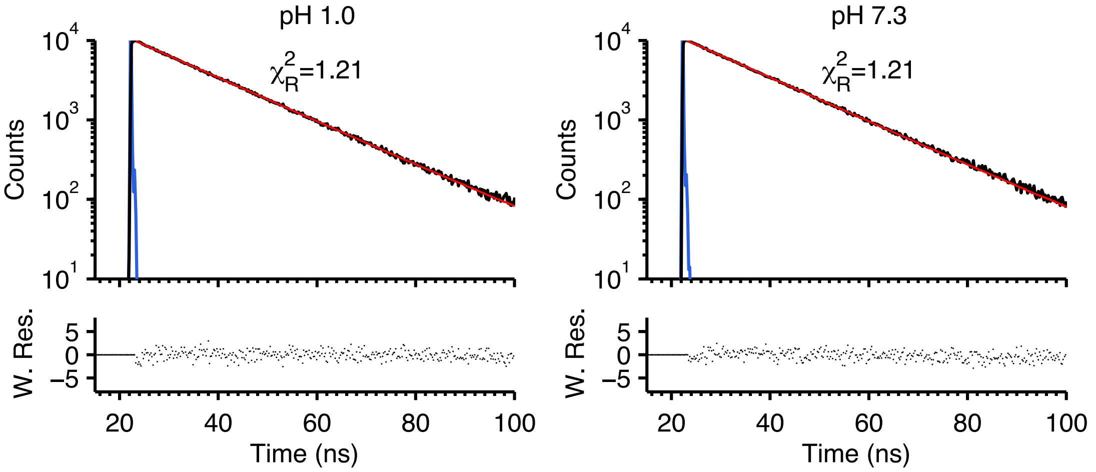

Figure S4. The effect of pH on the fluorescence lifetime of **DAOTA-Pr2**. pH 1.0 and 7.3 measurements were recorded in 0.1 M HCl and 10 mM lithium cacodylate buffer containing 100 mM KCl (pH 7.3). All fittings are to a mono-exponential decay model. Data traces, the instrument response and fittings are shown in black, blue and red respectively.

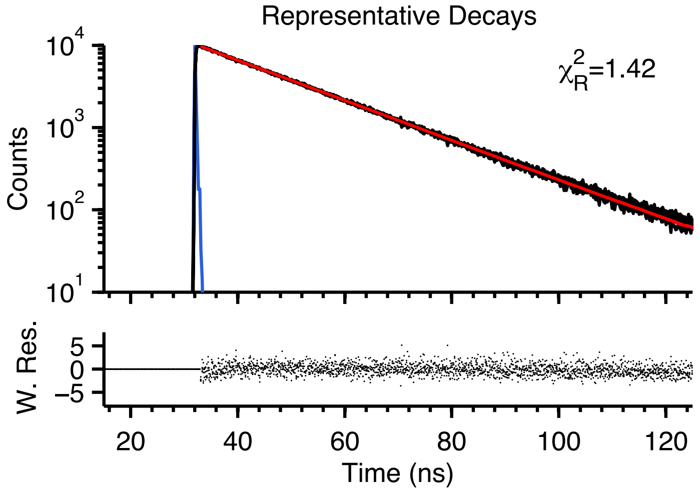

Figure S5. The effect of solvent polarity on the fluorescence lifetime of **DAOTA-Pr2** using global fitting. Only a mono-exponential decay was required to describe the decays in buffer systems (10 mM lithium cacodylate, pH 7.3 100 mM KCl) containing 20−66 % v/v 1,4- dioxane. For the representative decays plot, data traces, the instrument response and fittings are shown in black, blue and red respectively

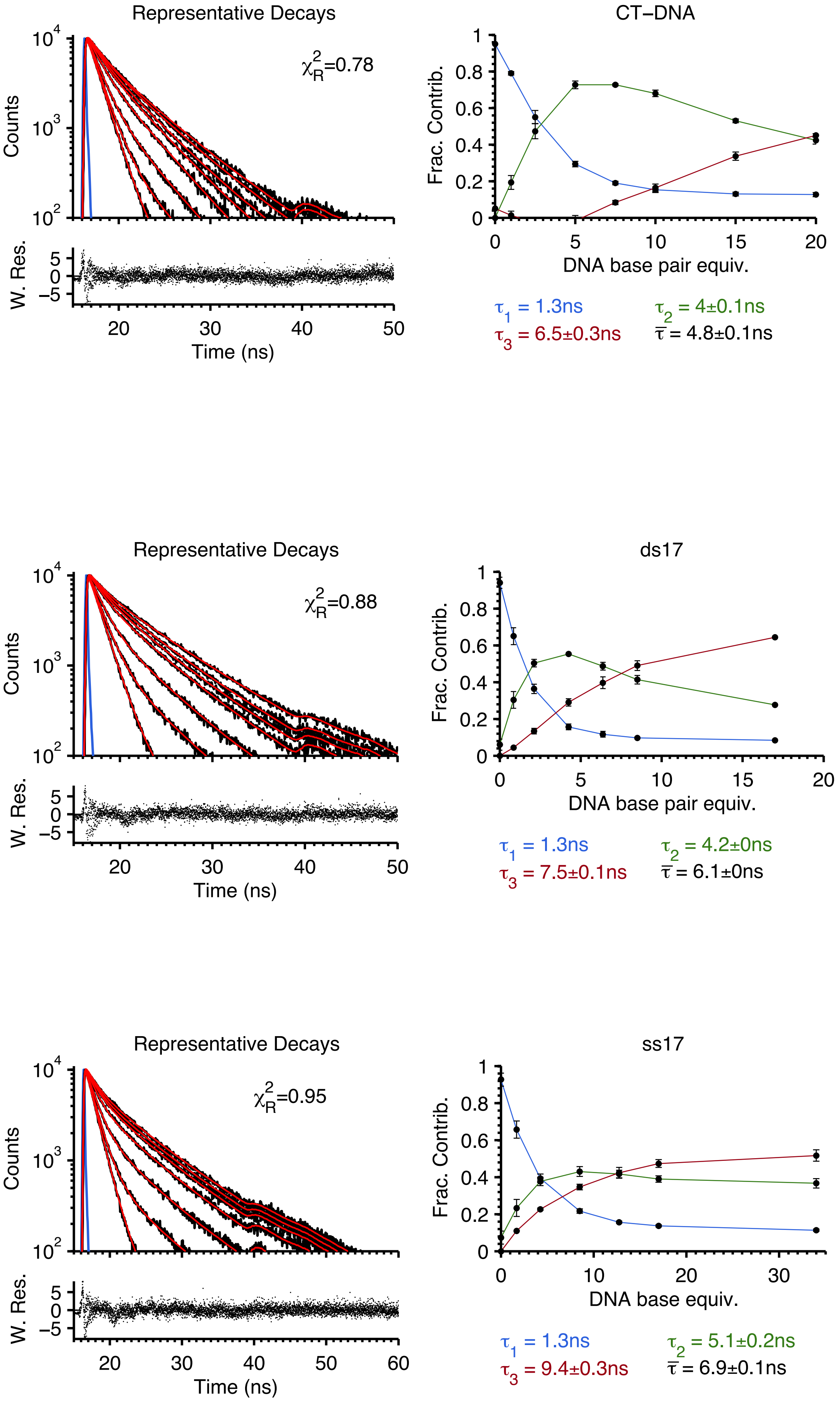

Representative Decays
χ^2_R=0.78
Counts
W. Res.
Time (ns)
CT–DNA
Frac. Contrib.
DNA base pair equiv.
τ_1 = 1.3ns
τ_2 = 4±0.1ns
τ_3 = 6.5±0.3ns
$\bar{\tau}$ = 4.8±0.1ns
Representative Decays
χ^2_R=0.88
Counts
W. Res.
Time (ns)
ds17
Frac. Contrib.
DNA base pair equiv.
τ_1 = 1.3ns
τ_2 = 4.2±0ns
τ_3 = 7.5±0.1ns
$\bar{\tau}$ = 6.1±0ns
Representative Decays
χ^2_R=0.95
Counts
W. Res.
Time (ns)
ss17
Frac. Contrib.
DNA base equiv.
τ_1 = 1.3ns
τ_2 = 5.1±0.2ns
τ_3 = 9.4±0.3ns
$\bar{\tau}$ = 6.9±0.1ns

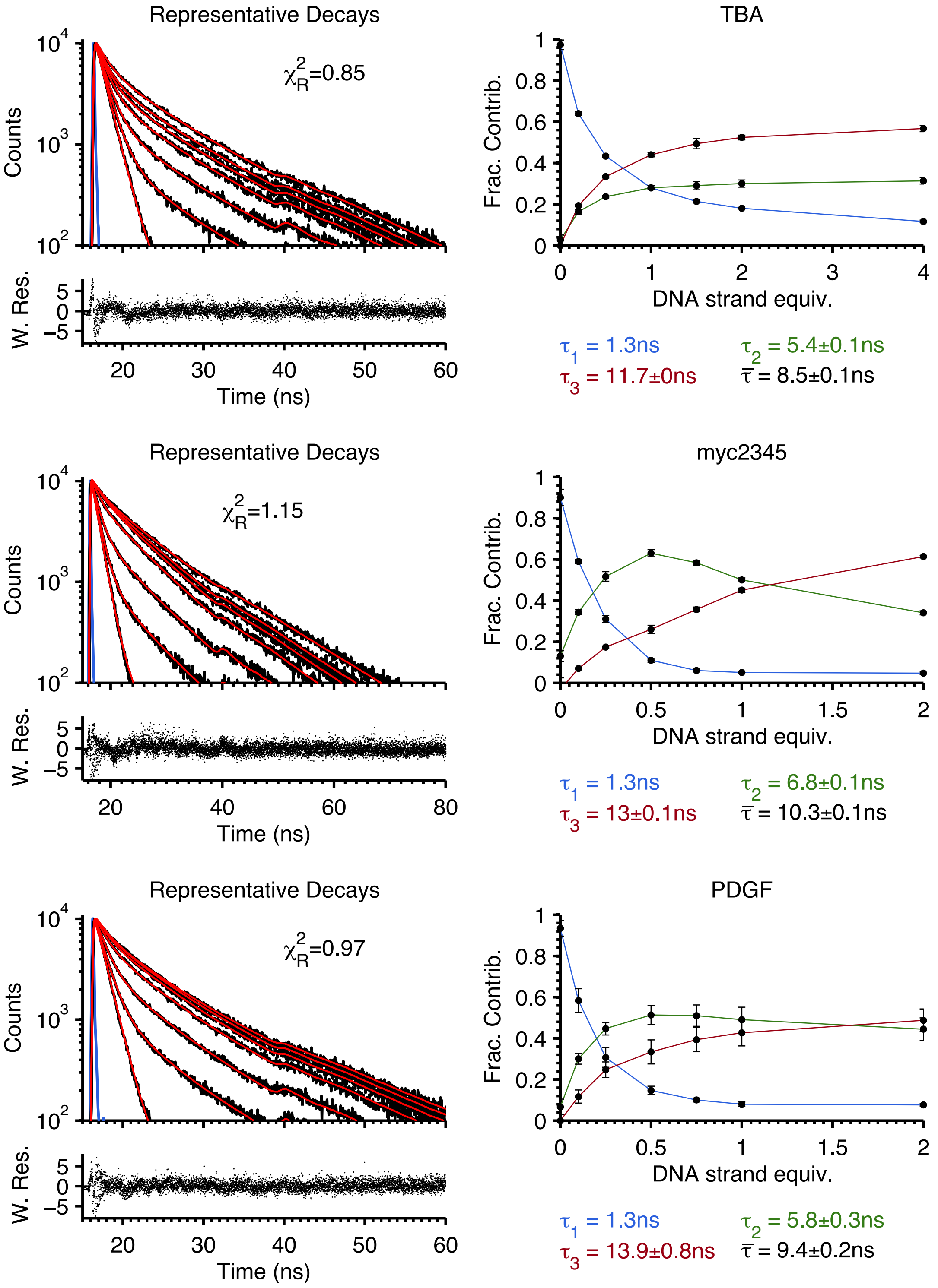

Figure S6. Fluorescence lifetime decay traces for **DAOTA-M2** (2 µM, 10 mM lithium cacodylate buffer pH 7.3, 100 mM KCl) upon titration of different DNA topologies and global fitting of τ_2 and τ_3 for all lifetime decay trace. For the representative decays plot, data traces, the instrument response and fittings are shown in black, blue and red respectively. For the fractional contribution plot, τ_1, τ_2 and τ_3 are colour coded with fractional contributions. Note that the lines are not fittings and are only present for clarity.

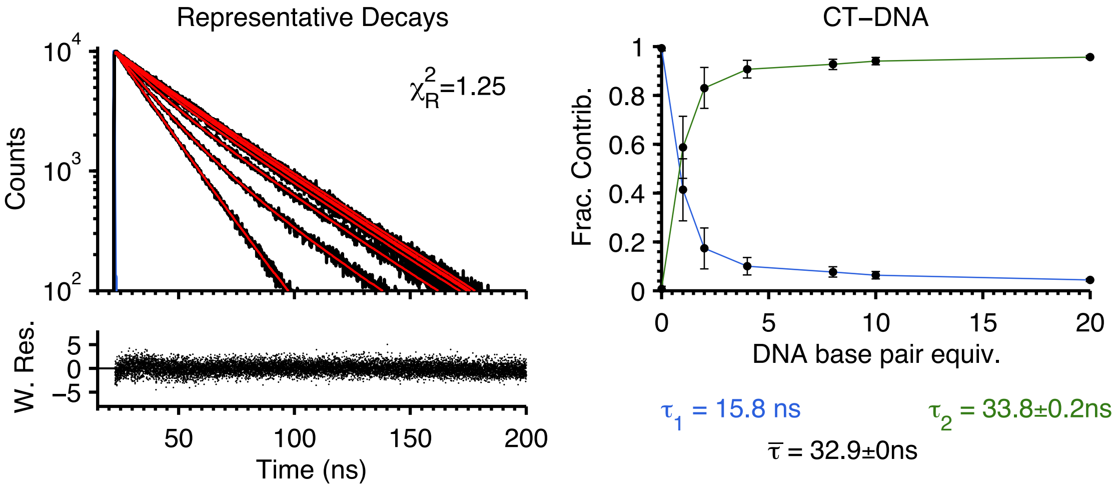
Representative Decays
CT−DNA
χ^2_R=1.25
Counts
W. Res.
Time (ns)
Frac. Contrib.
DNA base pair equiv.
τ_1 = 15.8 ns
τ_2 = 33.8±0.2ns
$\bar{\tau}$ = 32.9±0ns

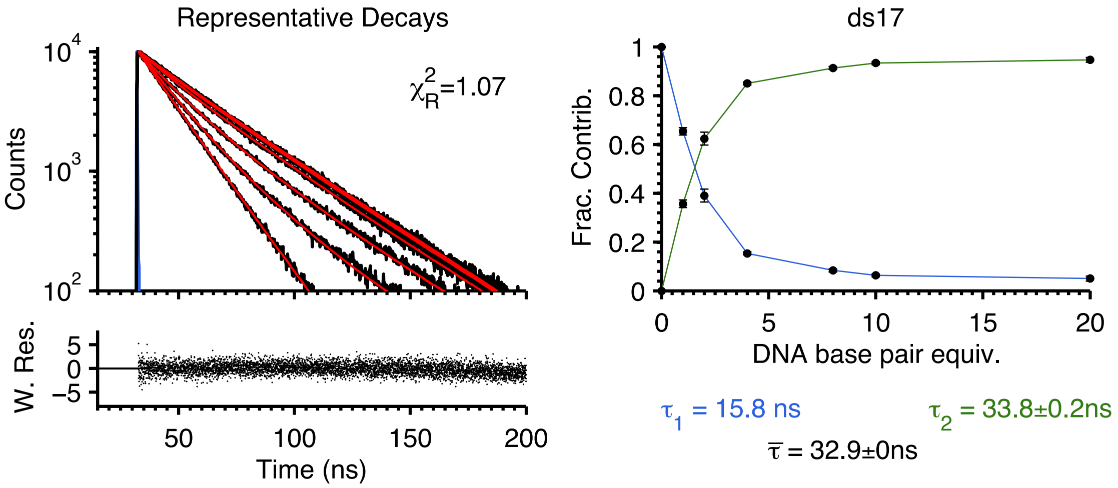
Representative Decays
ds17
χ^2_R=1.07
Counts
W. Res.
Time (ns)
Frac. Contrib.
DNA base pair equiv.
τ_1 = 15.8 ns
τ_2 = 33.8±0.2ns
$\bar{\tau}$ = 32.9±0ns

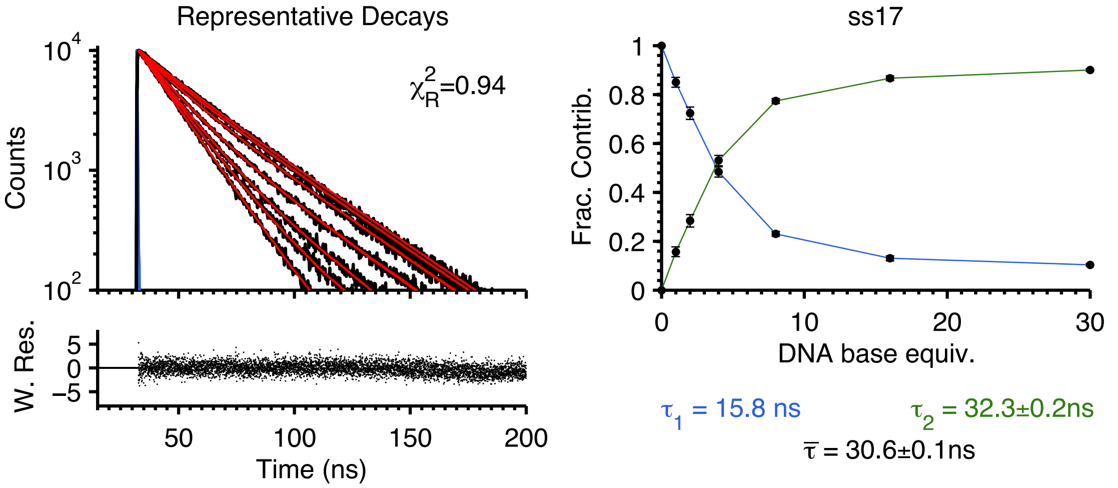
Representative Decays
ss17
χ^2_R=0.94
Counts
W. Res.
Time (ns)
Frac. Contrib.
DNA base equiv.
τ_1 = 15.8 ns
τ_2 = 32.3±0.2ns
$\bar{\tau}$ = 30.6±0.1ns

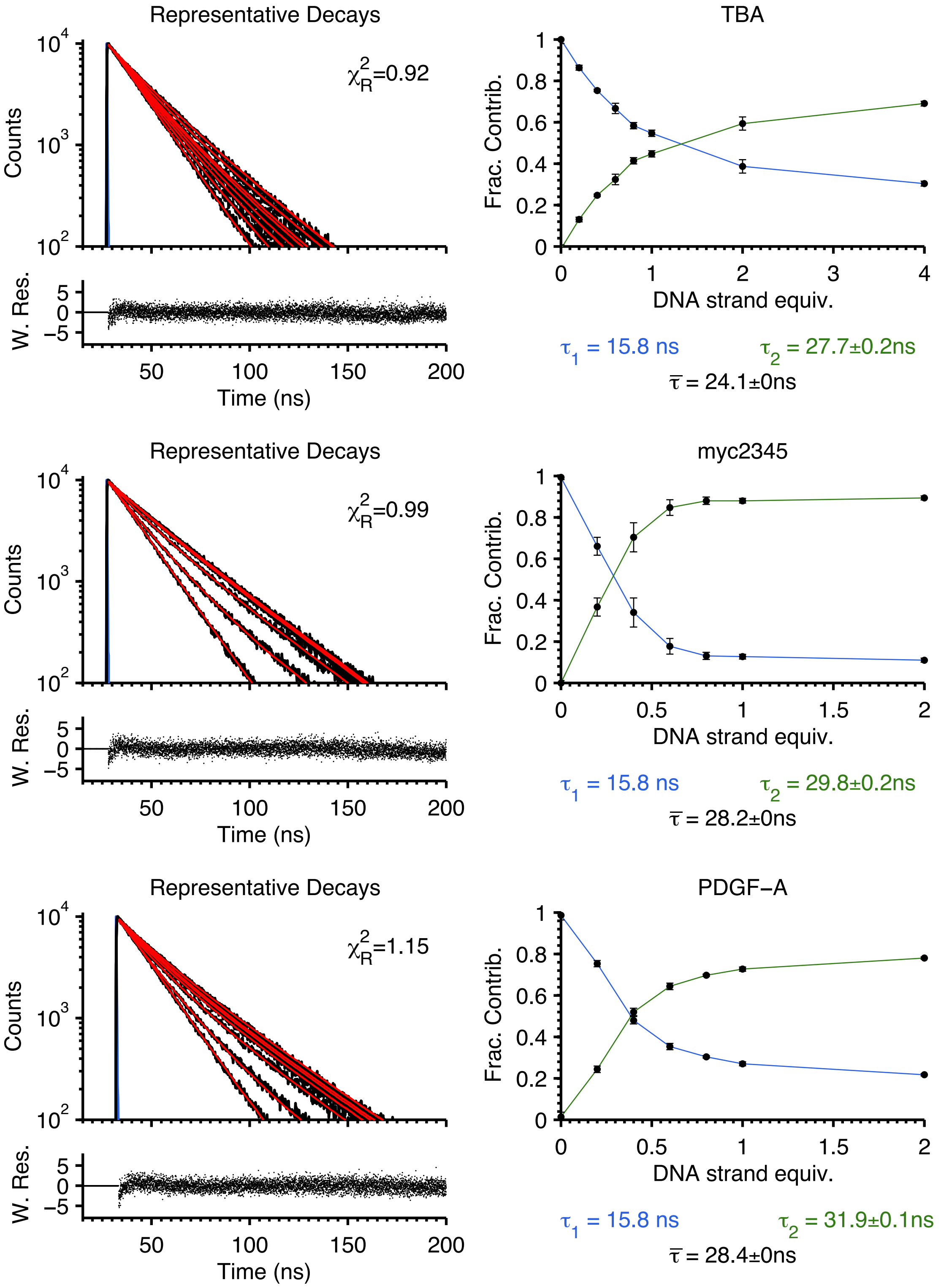

Figure S7. Fluorescence lifetime decay traces for **DAOTA-Pr2** (2 μM, 10 mM lithium cacodylate buffer pH 7.3, 100 mM KCl) upon titration of different DNA topologies and global fitting of τ_2 for all lifetime decay trace. For the representative decays plot, data traces, the instrument response and fittings are shown in black, blue and red respectively. For the fractional contribution plot, τ_1 and τ_2 are colour coded with fractional contributions. Note that the lines are not fittings and are only present for clarity.

CT-DNA

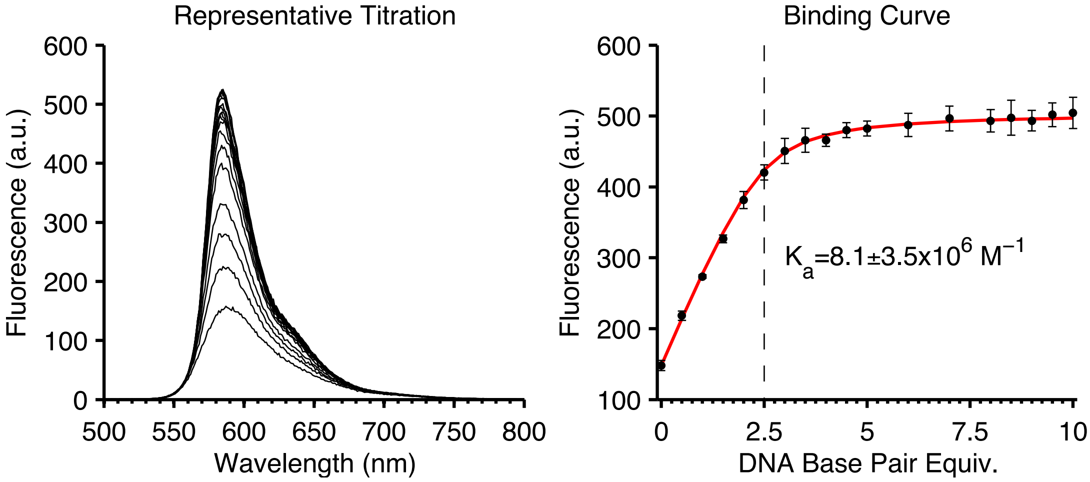

ds17

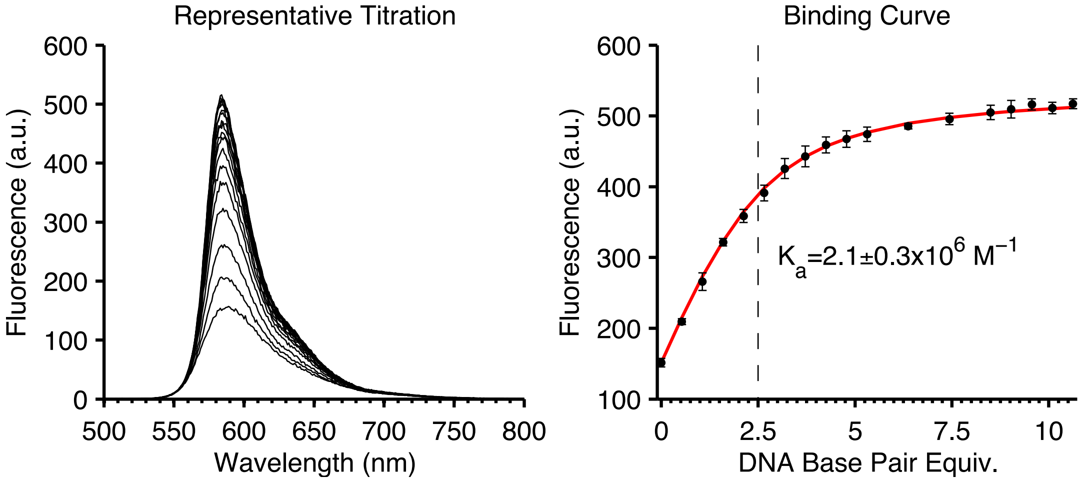

ss17

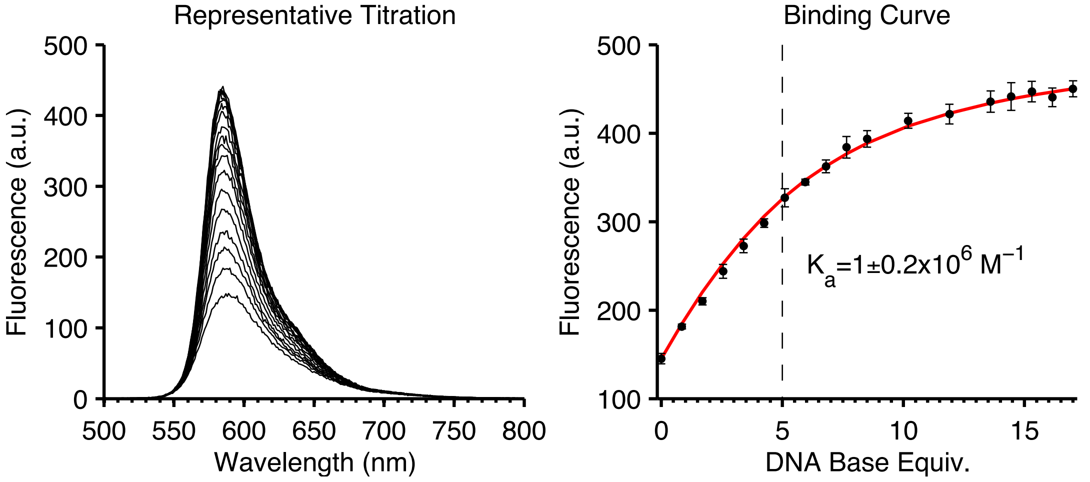

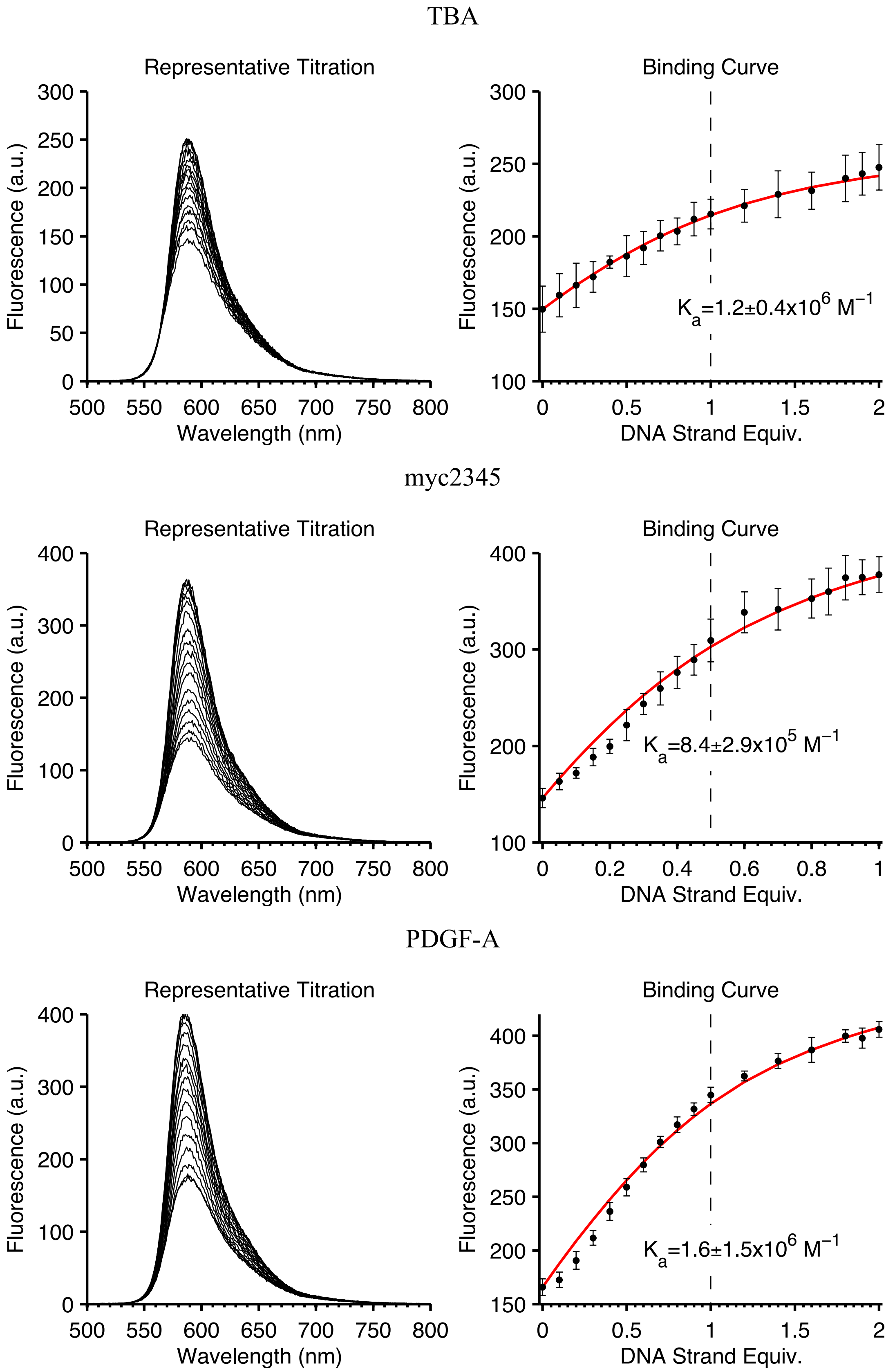

Figure S8. Emission titrations for **DAOTA-Pr2** with various DNA topologies. Error bars represent the standard deviation of three independent repeats. The average fitting is shown with no error bars (red) for clarity. Dashed lines indicate stoichiometry.

MeCN Solvation	E_{gas} (Hartree)	ZPE Corr. (Hartree)	$G_{gas,0\rightarrow 298K}$ Corr. (Hartree)	$G_{gas,corr.}$ (Hartree)	$\Delta G_{gas,EA}$ (Hartree)
TOTA, R	−955.232	0.230	0.190	−954.812	−0.204
TOTA, R^{-}	−955.428	0.226	0.186	−955.016	

MeCN Solvation	E_{gas} (Hartree)	$E_{solv.}$ (Hartree)	$\Delta G_{solv.}$ (Hartree)
TOTA, R	−955.232	-955.291	−0.059
TOTA, R^{-}	−955.428	-955.433	−0.005

$E(R/R^{-})$ (V)	$E(R/R^{-})$ *vs. NHE* (V)
4.07	-0.37

MeCN Solvation	E_{gas} (Hartree)	ZPE Corr. (Hartree)	$G_{gas,0\rightarrow 298K}$ Corr. (Hartree)	$G_{gas,corr.}$ (Hartree)	$\Delta G_{gas,EA}$ (Hartree)
TOTA, O^{+}	−954.809	0.226	0.186	-954.397	−0.415
TOTA, O	−955.232	0.230	0.190	-954.812	

MeCN Solvation	E_{gas} (Hartree)	$E_{solv.}$ (Hartree)	$\Delta G_{solv.}$ (Hartree)
TOTA, O^{+}	−954.809	-955.038	−0.229
TOTA, O	−955.232	-955.291	−0.059

$E(R/R^{-})$ (V)	$E(R/R^{-})$ *vs. NHE* (V)
6.68	2.24

MeCN Solvation	E_{gas} (Hartree)	ZPE Corr. (Hartree)	$G_{gas,0\rightarrow 298K}$ Corr. (Hartree)	$G_{gas,corr.}$ (Hartree)	$\Delta G_{gas,EA}$ (Hartree)
ADOTA-M, R	−1300.723	0.416	0.364	-1299.944	−0.189
ADOTA-M, R^{-}	−1300.904	0.412	0.359	-1300.133	

MeCN Solvation	E_{gas} (Hartree)	$E_{solv.}$ (Hartree)	$\Delta G_{solv.}$ (Hartree)
ADOTA-M, R	−1300.723	-1300.783	−0.060
ADOTA-M, R^{-}	−1300.904	-1300.914	−0.010

$E(R/R^{-})$ (V)	$E(R/R^{-})$ *vs. NHE* (V)
3.78	-0.66

MeCN Solvation	E_{gas} (Hartree)	ZPE Corr. (Hartree)	$G_{gas,0\rightarrow 298K}$ Corr. (Hartree)	$G_{gas,corr.}$ (Hartree)	$\Delta G_{gas,EA}$ (Hartree)
ADOTA-M, O^{+}	−1300.362	0.415	0.360	-1299.587	−0.356
ADOTA-M, O	−1300.723	0.416	0.364	-1299.944	

MeCN Solvation	E_{gas} (Hartree)	$E_{solv.}$ (Hartree)	$\Delta G_{solv.}$ (Hartree)
ADOTA-M, O^{+}	−1300.362	-1300.571	−0.209
ADOTA-M, O	−1300.723	-1300.783	−0.060

$E(R/R^{-})$ (V)	$E(R/R^{-})$ *vs. NHE* (V)
5.64	1.20

MeCN Solvation	E_{gas} (Hartree)	ZPE Corr. (Hartree)	$G_{gas,0\rightarrow 298K}$ Corr. (Hartree)	$G_{gas,corr.}$ (Hartree)	$\Delta G_{gas,EA}$ (Hartree)
DAOTA-M2, R	−1646.210	0.602	0.538	-1645.070	−0.177
DAOTA-M2, R^{-}	−1646.377	0.598	0.533	-1645.247	

MeCN Solvation	E_{gas} (Hartree)	$E_{solv.}$ (Hartree)	$\Delta G_{solv.}$ (Hartree)
DAOTA-M2, R	−1646.210	-1646.271	−0.061
DAOTA-M2,R^{-}	−1646.377	-1646.391	−0.014

$E(R/R^{-})$ (V)	$E(R/R^{-})$ *vs. NHE* (V)
3.52	-0.92

MeCN Solvation	E_{gas} (Hartree)	ZPE Corr. (Hartree)	$G_{gas,0\rightarrow 298K}$ Corr. (Hartree)	$G_{gas,corr.}$ (Hartree)	$\Delta G_{gas,EA}$ (Hartree)
DAOTA-M2,O^{+}	−1645.865	0.600	0.535	-1644.730	−0.340
DAOTA-M2, O	−1646.210	0.602	0.538	-1645.070	

MeCN Solvation	E_{gas} (Hartree)	$E_{solv.}$ (Hartree)	$\Delta G_{solv.}$ (Hartree)
DAOTA-M2,O^{+}	−1645.865	-1646.059	−0.193
DAOTA-M2, O	−1646.210	-1646.271	−0.061

$E(R/R^{-})$ (V)	$E(R/R^{-})$ *vs. NHE* (V)
5.66	1.22

Table S1. Energies calculated using the B3LYP/cc-pVTZ level of theory. IEFPCM acetonitrile solvation and unscaled ZPE were used.

H_2O Solvation	E_{gas} (Hartree)	ZPE Corr. (Hartree)	$G_{gas,0\rightarrow 298K}$ Corr. (Hartree)	$G_{gas,corr.}$ (Hartree)	$\Delta G_{gas,EA}$ (Hartree)
TOTA, R	-955.232	0.230	0.190	-954.812	-0.204
TOTA, R^{-}	-955.428	0.226	0.186	-955.016	

H_2O Solvation	E_{gas} (Hartree)	$E_{solv.}$ (Hartree)	$\Delta G_{solv.}$ (Hartree)
TOTA, R	-955.232	-955.292	-0.060
TOTA, R^{-}	-955.428	-955.433	-0.005

$E(R/R^{-})$ (V)	$E(R/R^{-})$ *vs. NHE* (V)
4.05	-0.39

H_2O Solvation	E_{gas} (Hartree)	ZPE Corr. (Hartree)	$G_{gas,0\rightarrow 298K}$ Corr. (Hartree)	$G_{gas,corr.}$ (Hartree)	$\Delta G_{gas,EA}$ (Hartree)
TOTA-M, O^{+}	-954.809	0.226	0.186	-954.397	-0.415
TOTA-M, O	-955.232	0.230	0.190	-954.812	

H_2O Solvation	E_{gas} (Hartree)	$E_{solv.}$ (Hartree)	$\Delta G_{solv.}$ (Hartree)
TOTA-M, O^{+}	-954.809	-955.041	-0.232
TOTA-M, O	-955.232	-955.292	-0.060

$E(R/R^{-})$ (V)	$E(R/R^{-})$ *vs. NHE* (V)
6.61	2.17

H_2O Solvation	E_{gas} (Hartree)	ZPE Corr. (Hartree)	$G_{gas,0\rightarrow 298K}$ Corr. (Hartree)	$G_{gas,corr.}$ (Hartree)	$\Delta G_{gas,EA}$ (Hartree)
ADOTA-M, R	-1300.723	0.416	0.364	-1299.944	-0.189
ADOTA-M, R^{-}	-1300.904	0.412	0.359	-1300.133	

H_2O Solvation	E_{gas} (Hartree)	$E_{solv.}$ (Hartree)	$\Delta G_{solv.}$ (Hartree)
ADOTA-M, R	-1300.723	-1300.784	-0.061
ADOTA-M, R^-	-1300.904	-1300.914	-0.010

$E(R/R^-)$ (V)	$E(R/R^-)$ *vs. NHE* (V)
3.76	-0.68

H_2O Solvation	E_{gas} (Hartree)	ZPE Corr. (Hartree)	$G_{gas,0\rightarrow 298K}$ Corr. (Hartree)	$G_{gas,corr.}$ (Hartree)	$\Delta G_{gas,EA}$ (Hartree)
ADOTA-M, O^+	-1300.362	0.415	0.360	-1299.587	-0.356
ADOTA-M, O	-1300.723	0.416	0.364	-1299.944	

H_2O Solvation	E_{gas} (Hartree)	$E_{solv.}$ (Hartree)	$\Delta G_{solv.}$ (Hartree)
ADOTA-M, O^+	-1300.362	-1300.575	-0.213
ADOTA-M, O	-1300.723	-1300.784	-0.061

$E(R/R^-)$ (V)	$E(R/R^-)$ *vs. NHE* (V)
5.57	1.13

H_2O Solvation	E_{gas} (Hartree)	ZPE Corr. (Hartree)	$G_{gas,0\rightarrow 298K}$ Corr. (Hartree)	$G_{gas,corr.}$ (Hartree)	$\Delta G_{gas,EA}$ (Hartree)
ADOTA-M + H^+, R	-1301.009	0.430	0.378	-1300.200	-0.277
ADOTA-M + H^+, R^-	-1301.276	0.426	0.373	-1300.477	

H_2O Solvation	E_{gas} (Hartree)	$E_{solv.}$ (Hartree)	$\Delta G_{solv.}$ (Hartree)
ADOTA-M + H^+, R	-1301.009	-1301.228	-0.220
ADOTA-M + H^+, R^-	-1301.276	-1301.363	-0.087

$E(R/R^-)$ (V)	$E(R/R^-)$ *vs. NHE* (V)
3.90	-0.54

H_2O Solvation	E_{gas} (Hartree)	ZPE Corr. (Hartree)	$G_{gas,0\rightarrow 298K}$ Corr. (Hartree)	$G_{gas,corr.}$ (Hartree)	$\Delta G_{gas,EA}$ (Hartree)
ADOTA-M + H^+, O^+	-1300.525	0.427	0.375	-1299.722	-0.478
ADOTA-M + H^+, O	-1301.009	0.430	0.378	-1300.200	

H_2O Solvation	E_{gas} (Hartree)	$E_{solv.}$ (Hartree)	$\Delta G_{solv.}$ (Hartree)
ADOTA-M + H^+, O^+	-1300.525	-1300.991	-0.466
ADOTA-M + H^+, O	-1301.009	-1301.228	-0.220

$E(R/R^-)$ (V)	$E(R/R^-)$ *vs. NHE* (V)
6.30	1.86

H_2O Solvation	E_{gas} (Hartree)	ZPE Corr. (Hartree)	$G_{gas,0\to298K}$ Corr. (Hartree)	$G_{gas,corr.}$ (Hartree)	$\Delta G_{gas,EA}$ (Hartree)
DAOTA-M2, R	-1646.210	0.602	0.538	-1645.070	-0.177
DAOTA-M2,R^-	-1646.377	0.598	0.533	-1645.247	

H_2O Solvation	E_{gas} (Hartree)	$E_{solv.}$ (Hartree)	$\Delta G_{solv.}$ (Hartree)
DAOTA-M2, R	-1646.210	-1646.272	-0.062
DAOTA-M2,R^-	-1646.377	-1646.392	-0.014

$E(R/R^-)$ (V)	$E(R/R^-)$ *vs. NHE* (V)
3.50	-0.94

H_2O Solvation	E_{gas} (Hartree)	ZPE Corr. (Hartree)	$G_{gas,0\to298K}$ Corr. (Hartree)	$G_{gas,corr.}$ (Hartree)	$\Delta G_{gas,EA}$ (Hartree)
DAOTA-M2, O^+	-1645.865	0.600	0.535	-1644.730	-0.340
DAOTA-M2, O	-1646.210	0.602	0.538	-1645.070	

H_2O Solvation	E_{gas} (Hartree)	$E_{solv.}$ (Hartree)	$\Delta G_{solv.}$ (Hartree)
DAOTA-M2, O^+	-1645.865	-1646.062	-0.197
DAOTA-M2, O	-1646.210	-1646.272	-0.062

$E(R/R^-)$ (V)	$E(R/R^-)$ *vs. NHE* (V)
5.59	1.15

H_2O Solvation	E_{gas} (Hartree)	ZPE Corr. (Hartree)	$G_{gas,0\to298K}$ Corr. (Hartree)	$G_{gas,corr.}$ (Hartree)	$\Delta G_{gas,EA}$ (Hartree)
DAOTA-M2 + H^+, R	-1646.502	0.616	0.552	-1645.334	-0.260
DAOTA-M2 + H^+, R^-	-1646.753	0.612	0.547	-1645.594	

H_2O Solvation	E_{gas} (Hartree)	$E_{solv.}$ (Hartree)	$\Delta G_{solv.}$ (Hartree)
DAOTA-M2 + H^+, R	-1646.502	-1646.717	-0.215
DAOTA-M2 + H^+, R^-	-1646.753	-1646.841	-0.088

$E(R/R^-)$ (V)	$E(R/R^-)$ *vs. NHE* (V)
3.63	-0.81

H_2O Solvation	E_{gas} (Hartree)	ZPE Corr. (Hartree)	$G_{gas,0\to298K}$ Corr. (Hartree)	$G_{gas,corr.}$ (Hartree)	$\Delta G_{gas,EA}$ (Hartree)
DAOTA-M2 + H^+, O^+	-1646.090	0.615	0.551	-1644.924	-0.410
DAOTA-M2 + H^+, O	-1646.502	0.616	0.552	-1645.334	

H_2O Solvation	E_{gas} (Hartree)	$E_{solv.}$ (Hartree)	$\Delta G_{solv.}$ (Hartree)
DAOTA-M2 + H^+, O^+	-1646.090	-1646.508	-0.417
DAOTA-M2 + H^+, O	-1646.502	-1646.717	-0.215

$E(R/R^-)$ (V)	$E(R/R^-)$ *vs. NHE* (V)
5.64	1.20

H_2O Solvation	E_{gas} (Hartree)	ZPE Corr. (Hartree)	$G_{gas,0\rightarrow 298K}$ Corr. (Hartree)	$G_{gas,corr.}$ (Hartree)	$\Delta G_{gas,EA}$ (Hartree)
DAOTA-M2 + 2 H^+, R	-1646.739	0.630	0.567	-1645.542	-0.344
DAOTA-M2 + 2 H^+, R^-	-1647.074	0.627	0.562	-1645.886	

H_2O Solvation	E_{gas} (Hartree)	$E_{solv.}$ (Hartree)	$\Delta G_{solv.}$ (Hartree)
DAOTA-M2 + 2 H^+, R	-1646.739	-1647.161	-0.422
DAOTA-M2 + 2 H^+, R^-	-1647.074	-1647.291	-0.217

$E(R/R^-)$ (V)	$E(R/R^-)$ *vs. NHE* (V)
3.75	-0.69

H_2O Solvation	E_{gas} (Hartree)	ZPE Corr. (Hartree)	$G_{gas,0\rightarrow 298K}$ Corr. (Hartree)	$G_{gas,corr.}$ (Hartree)	$\Delta G_{gas,EA}$ (Hartree)
DAOTA-M2 + 2 H^+, O^+	-1646.195	0.627	0.563	-1645.005	-0.537
DAOTA-M2 + 2 H^+, O	-1646.739	0.630	0.567	-1645.542	

H_2O Solvation	E_{gas} (Hartree)	$E_{solv.}$ (Hartree)	$\Delta G_{solv.}$ (Hartree)
DAOTA-M2 + 2 H^+, O^+	-1646.195	-1646.931	-0.735
DAOTA-M2 + 2 H^+, O	-1646.739	-1647.161	-0.422

$E(R/R^-)$ (V)	$E(R/R^-)$ *vs. NHE* (V)
6.08	1.64

Table S2. Energies calculated using the B3LYP/cc-pVTZ level of theory. IEFPCM aqueous solvation and unscaled ZPE were used.

Compound	Geometry	Excitation Energy (eV)	Oscillator Strength (f)	Composition (CI Co-efficient)
ADOTA-M	Ground	2.92	0.2417	HOMO→LUMO (0.69)<br>HOMO-1→LUMO (0.10)
	Excited	2.72	0.3145	HOMO→LUMO (0.67)<br>HOMO-1→LUMO (-0.20)
DOATA-M2	Ground	2.76	0.4173	HOMO→LUMO (0.70)
	Excited	2.67	0.3934	HOMO→LUMO (0.70)

Table S3. First excited state ($S_0 \rightarrow S_1$) TD-DFT energies, oscillator strengths and molecular orbitals at the CAM-B3LYP/6-311G+(2d,p) level of theory using IEFPCM aqueous solvation.

Supplementary information – X-ray crystallography

The X-ray crystal structure of ADOTA-M. The PF_6 anion in the structure of **ADOTA-M** (Fig. S9) was found to be disordered. Three orientations were identified, of *ca*. 49, 28 and 23% occupancy, their geometries were optimised, the thermal parameters of adjacent atoms were restrained to be similar, and only the atoms of the major occupancy orientation were refined anisotropically (the remainder were refined isotropically).

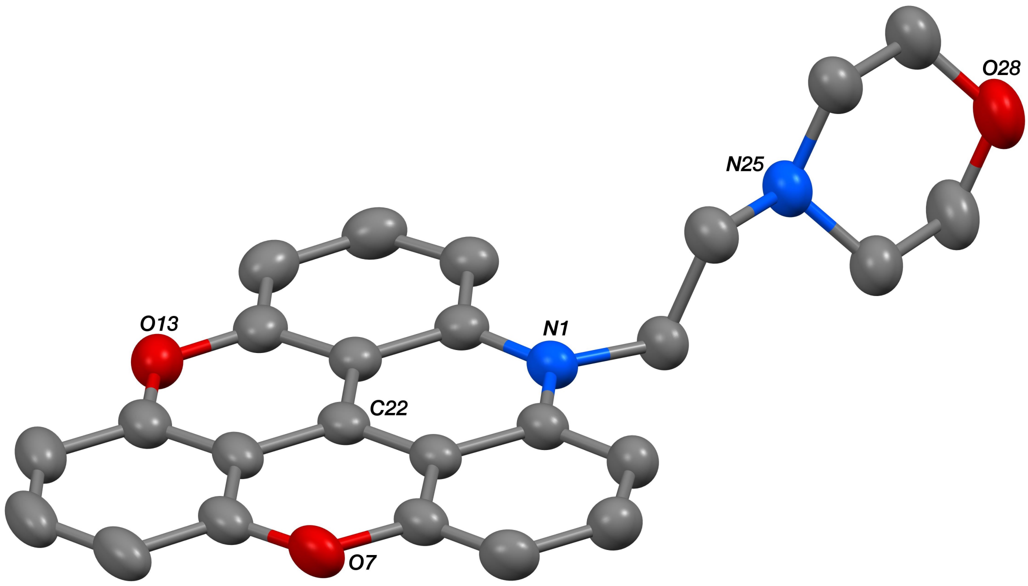

Figure S9. The crystal structure of **ADOTA-M** (50% probability ellipsoids).

The X-ray crystal structure of reduced TMPA-M. Crystals of reduced **TMPA-M** were obtained as a by-product during **TMPA-M** work up. In contrast to the planarity seen for the fused ring system in the structure of **ADOTA-M**, the acridine ring system in the structure of **TMPA-M** (Fig. 6) is significantly folded, the two outer aryl rings being inclined by *ca*. 23°. The –CH_2–CH_2–morpholino unit was unfortunately found to be disordered in the crystal structure (see supporting information for more details) so analysis of its geometry needs to be treated with caution. For the major occupancy orientation (*ca*. 82% occupancy) the N–CH_2–CH_2–N linkage between the acridine and morpholino ring systems is much closer to an ideal *anti* conformation than seen for **ADOTA–M**, the torsion angle being 170.9(2)° *cf.* 152.05(19)° in **ADOTA-M**. As expected this "straightening" of the linkage is associated with a lengthening of the N···N separation, which is 3.784(3) Å here *cf.* 3.712(3) Å in **ADOTA-M**. There are no noteworthy intermolecular interactions.

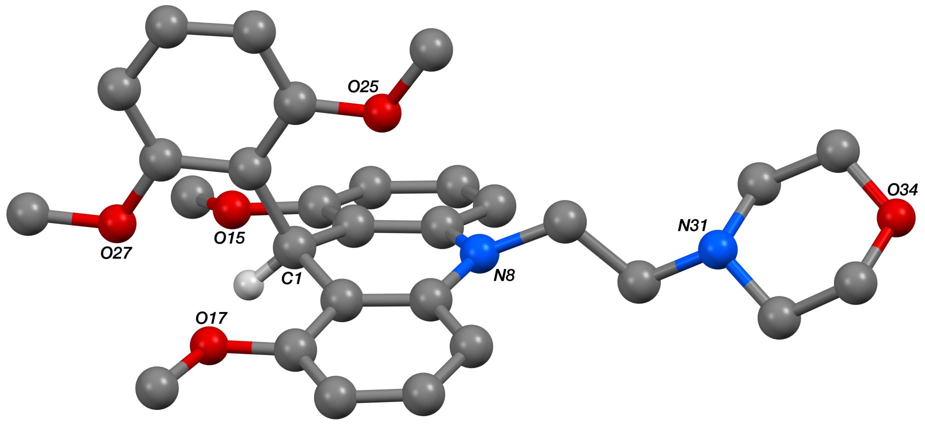

Figure S10. The X-ray crystal structure of reduced **TMPA-M**.
